# Microglia-specific knock-down of Bmal1 improves memory and protects mice from high fat diet-induced obesity

**DOI:** 10.1038/s41380-021-01169-z

**Published:** 2021-05-28

**Authors:** Xiao-Lan Wang, Sander Kooijman, Yuanqing Gao, Laura Tzeplaeff, Brigitte Cosquer, Irina Milanova, Samantha E. C. Wolff, Nikita Korpel, Marie-France Champy, Benoit Petit-Demoulière, Isabelle Goncalves Da Cruz, Tania Sorg-Guss, Patrick C. N. Rensen, Jean-Christophe Cassel, Andries Kalsbeek, Anne-Laurence Boutillier, Chun-Xia Yi

**Affiliations:** 1 Université de Strasbourg, Laboratoire de Neuroscience Cognitives et Adaptatives (LNCA), Strasbourg, France; 2 Department of Endocrinology and Metabolism, Amsterdam University Medical Centres (UMC), University of Amsterdam, Amsterdam, The Netherlands; 3 Laboratory of Endocrinology, Amsterdam University Medical Centres (UMC), University of Amsterdam, Amsterdam Gastroenterology & Metabolism, Amsterdam, The Netherlands; 4 Department of Medicine, Divison of Endocrinology, and Einthoven Laboratory for Experimental Vascular Medicine, Leiden University Medical Center, Leiden, The Netherlands; 5 CNRS UMR 7364, LNCA, Strasbourg, France; 6 Netherlands Institute for Neuroscience, an Institute of the Royal Netherlands Academy of Arts and Sciences, Amsterdam, The Netherlands; 7 PHENOMIN-ICS, Institut Clinique de la souris, CNRS, UMR7104, Illkirch, France; 8 INSERM, U964, Illkirch, France; 9 Université de Strasbourg, Strasbourg, France

**Keywords:** Neuroscience, Cell biology

## Abstract

Microglia play a critical role in maintaining neural function. While microglial activity follows a circadian rhythm, it is not clear how this intrinsic clock relates to their function, especially in stimulated conditions such as in the control of systemic energy homeostasis or memory formation. In this study, we found that microglia-specific knock-down of the core clock gene, *Bmal1*, resulted in increased microglial phagocytosis in mice subjected to high-fat diet (HFD)-induced metabolic stress and likewise among mice engaged in critical cognitive processes. Enhanced microglial phagocytosis was associated with significant retention of pro-opiomelanocortin (POMC)-immunoreactivity in the mediobasal hypothalamus in mice on a HFD as well as the formation of mature spines in the hippocampus during the learning process. This response ultimately protected mice from HFD-induced obesity and resulted in improved performance on memory tests. We conclude that loss of the rigorous control implemented by the intrinsic clock machinery increases the extent to which microglial phagocytosis can be triggered by neighboring neurons under metabolic stress or during memory formation. Taken together, microglial responses associated with loss of Bmal1 serve to ensure a healthier microenvironment for neighboring neurons in the setting of an adaptive response. Thus, microglial Bmal1 may be an important therapeutic target for metabolic and cognitive disorders with relevance to psychiatric disease.

## Introduction

Microglia are resident brain macrophages that are responsible for elimination of apoptotic cells, cellular debris, and invading pathogens. They can regulate synaptic remodeling to optimize the microenvironment for neuronal survival and function [1, 2]. Microglial phagocytosis maintains the hypothalamic neural circuitry that controls energy homeostasis and also shapes hippocampal neuronal synapses so that they can establish mature connections [3, 4]. Aberrant microglial phagocytosis is associated with obesity, neurodegenerative disorders, and psychiatric disease [5, 6].

Microglial activity follows a circadian rhythm [7–9], which is an internal timekeeping system that coordinates physiological processes and behaviors in accordance with day/night cycles [10, 11]. In mammals, the core mechanism of the molecular clock machinery in nearly every cell type includes autoregulatory transcriptional and translational feedback loops that are controlled by the actions of clock genes [12]. Molecular clocks drive circadian rhythms that modulate gene expression and cell function [13, 14]. Among the most prominent of these regulatory factors, the transcriptional activator, the Brain and Muscle Arnt-like 1 (Bmal1)/Clock complex promotes the expression of Period (*Per1*, *Per2*) and Cryptochrome (*Cry1*, *Cry2*) genes via binding interactions with their respective E-box promoter elements. The Per/Cry complex then negatively regulates its own transcription by inhibiting the activity of the Bmal1/Clock complex [12, 15]. Disruption of the clock machinery has a significant impact on mammalian physiology [16]. For example, skeletal muscle-specific deficiency of the *Clock* gene results in disturbed nutrient utilization and leads to metabolic disorders [17, 18]. Hippocampal-dependent cognitive performance is also controlled by the circadian clock [19]. However, specific functions regulated by the microglial circadian clock and its role with respect to the control of energy homeostasis and cognition have not yet been clarified.

Previous studies revealed that mice with a global knockout of the core clock gene *Bmal1*, are completely devoid of circadian rhythms in light/dark and constant dark conditions and show a variety of phenotypic abnormalities, among which age-related astrogliosis in the cortex and hippocampus, degeneration of synaptic terminals, and impaired cortical functional connectivity [20, 21]. In cultured microglial cells, Bmal1 deficiency resulted in diminished expression of interleukin-6 (*Il6*) upon lipopolysaccharide challenging [22]. However, this previous study did not address the impact of Bmal1 deletion on microglial phagocytic capacity. Microglial phagocytosis in the adult mouse brain is subject to region-specific differences that depend on neuronal activity [23]. In this study, we generated mice with microglia-specific Bmal1-deficiency to explore cell-autonomous microglia-related functions, particularly regarding how the Bmal1-regulated phagocytosis is involved in stress-coping capacity by neurocircuitries in the hypothalamus and hippocampus. We also performed experiments that addressed the impact of microglial Bmal1 deletion on systemic energy homeostasis and on processes associated with learning and memory.

## Materials and methods

### Animals

Two lines of transgenic mice (*Cx3cr1*^CreER^ mice [24, 25], stock no. 021160 and *Bmal1*^lox/lox^ mice [26], stock no. 007668) were obtained from the Jackson Laboratory (Bar Harbor, ME, USA) and crossed in this study. The *Cx3cr1*^CreER^ mice harbor a tamoxifen-inducible Cre recombinase that is fused with an enhanced yellow fluorescent protein (EYFP) attached to the chemokine (CX_3_C motif) receptor 1 (*Cx3cr1*) promoter. The cellular localization of Cre was examined by the colocalization of EYFG with Iba1 immunoreactivity in our study. Almost all EYFP positive cells were Iba1-positive microglia, suggesting that the Cre is specifically expressed in microglia (Fig. S1A). To excise the loxP-flanked *Bmal1* sequences via Cre-mediated recombination, all 8–10-week-old mice were treated with tamoxifen (20 mg/ml; T5648, Sigma-Aldrich) in corn oil (S5007, Sigma-Aldrich) via intraperitoneal (i.p.) injection, once each day for 3 days at a dose of 100 μl per injection. Mice that were *Bmal1* lox-homozygous and Cre-positive (*Bmal1*^lox/lox^-*Cx3cr1*^CreER^) were used as the microglia^*Bmal1*-KD^ model. Cre-positive, *Bmal1* wild-type mice served as littermate controls (Ctrls; *Cx3cr1*^CreER^) (Knocking down validation and experimental groups allocation see Supplementary information).

### Metabolic phenotype

After tamoxifen injection at 8–10 weeks of age, mice were fed either standard chow or a high-fat diet (HFD; Research Diets, Inc., New Brunswick, NJ, USA, cat. no. D12492). Body weight was measured weekly after tamoxifen injections. Food intake was measured daily for 5 days at ZT0 and ZT12 after 4 weeks on each diet. Energy expenditure, as well as physical activity, respiratory exchange ratio (RER), and heat production were evaluated by indirect calorimetry using the Phenomaster system (TSE Systems, Phenomaster/Labmaster, Bad Homburg, Germany) after 5 weeks on the HFD (see Supplementary information).

### Cognitive phenotype

To test the learning and memory capacities, male microglia^*Bmal1*-KD^ and Ctrl mice performed the Morris water maze (MWM) and novel object recognition (NOR) tests 2 weeks after the tamoxifen injections. A different group of mice was used in each specific behavioral experiment as described in Fig. 3A–J (see Supplementary information).

### Labeling and counting dendritic spines after Golgi staining

Mice were subjected to a 4-day-training in the MWM and killed 4 days after the last training. Golgi staining was performed for labeling and counting dendritic spines (see Supplementary information).

### Profiling microglial phagocytic capacity and gene and protein expressions

To investigate microglial phagocytic capacity, primary microglial cells were prepared as described previously [27]. Fluoresbrite^®^ Polychromatic Red Microspheres (1.0 µm, 18660-5, polysciences) were used to treat the cells and the average of microspheres per cell was measured (see Supplementary information). To analyze microglial gene and protein expression, 3 weeks after the tamoxifen injections, mice were decapitated for extraction of brain tissue. Microglia were isolated for RT-PCR and western blot studies (see Supplementary information).

### Characterizing neurons and microglia in the hypothalamus and hippocampus

At the end of metabolic or cognitive studies, mouse brain tissues were obtained after perfusion-fixation. Brain sections were processed for immunohistochemical or immunofluorescence staining. Bright field or fluorescent confocal images were acquired accordingly and images were analyzed using Image J or Imaris software with 3D reconstruction (Bitplane AG, Zurich, Switzerland) (Supplementary information).

### Statistical analysis

Experimenters were blinded to the mouse genotype during behavioral testing and imaging analysis. Where applicable, statistical analyses were performed using two-tailed unpaired *t*-tests and two-way analysis of variance (ANOVA) with GraphPad Prism 8 (San Diego, CA, USA). One-way ANOVA was used to assess the effect of time on microglial gene expression in the C57BL/6J wild-type mice. The daily rhythms associated with gene expression in microglia in wild-type mice were evaluated by cosinor analysis using SigmaPlot 12.0 software (SPSS Inc., Chicago, IL, USA) [28]. Data were fitted to the following regression: *y* = A + B × cos(2π [x − C]/24) where A is the mean level, B is the amplitude, and C is the acrophase of the fitted rhythm. An overall *p* value (main *p* value, *P*m) was considered as indicating 24-h-rhythmicity. The variation of data between the groups that compared is similar. All data are presented as mean ± standard error of the mean (s.e.m.) with statistical significance indicated by *P* < 0.05.

## Results

### Microglia-specific knock-down of Bmal1 deregulates the expression of clock-related genes

**Fig. 1:**
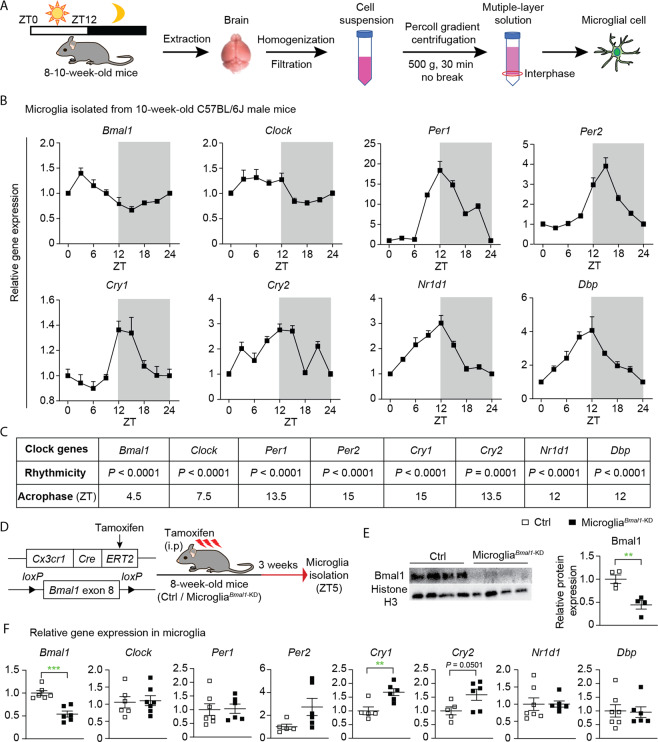
Specific knock-down of microglial *Bmal1* (microglia^*Bmal1*-KD^) alters the expression of clock genes in adult male mice. **A** Strategy used to isolate microglia in this study. ZT0 = lights on; ZT12 = lights off. **B** Relative expression of clock genes in microglia isolated from brains of wild-type C57BL/6J male mice. Shown are data obtained from microglia isolated every 3 h from ZT0 through ZT21 (*n* = 8 mice per group); data shown for points ZT0 and ZT24 are from the same samples. **C** Statistical analysis of rhythmic expression and acrophase determined for each of the clock genes; *P* < 0.05 was considered as representing significant rhythmicity. **D** Experimental strategy used for postnatal deletion of *Bmal1* specifically in microglia and the time course for microglial isolation. **E** Representative images and quantification of western blots documenting immunoreactive Bmal1 and Histone H3 in isolated and CD11b-affinity purified microglia (*n* = 4 mice per group). **F** Expression of clock genes in microglia isolated from the brain of Ctrl and microglia^*Bmal1*-KD^ mice at 3 weeks after the tamoxifen injections (*n* = 5–7 mice per group). Data are presented as means ± s.e.m. Green-colored * indicates a genotype effect; **P < 0.01, ***P < 0.001.

To examine the cell-specific expression of clock-related genes, we isolated microglia from brain tissue of wild-type C57BL/6J mice every 3 h following lights on (i.e., ZT0; Fig. 1A). We found that the expression of clock genes in mouse microglial cells follows a distinct circadian rhythm (Fig. 1B, C). In order to study the functions of Bmal1 in microglia of adult mice, we generated microglia^*Bmal1*-KD^ mice that harbor a microglia-specific deletion of this gene. Specifically, the microglia^*Bmal1*-KD^ strain was generated by crossing *Bmal1*^lox/lox^ mice with *Cx3cr1*^CreER^ mice [24, 25]; *Bmal1*-sufficient *Cx3cr1*^CreER^ mice were used as controls (Ctrls; Fig. 1D). Three weeks after administration of tamoxifen, we observed marked reductions in immunoreactive Bmal1 on western blots of microglia isolated from microglia^*Bmal1*-KD^ mice compared with that detected in Ctrls (Fig. 1E and Fig. S1B, C). These results documented the efficient tamoxifen-mediated knock-down of microglial *Bmal1* expression in the microglia^*Bmal1*-KD^ mouse strain. Furthermore, we found a significant reduction of *Bmal1* gene expression while both *Cry1* and *Cry2* were increased in microglia isolated from male microglia^*Bmal1*-KD^ mice (Fig. 1F). By contrast, in female mice of this strain only expression of the D site albumin promoter binding protein gene (*Dbp*) was significantly diminished (Fig. S1D).

### Microglial deficiency of Bmal1 limits HFD-induced obesity

**Fig. 2:**
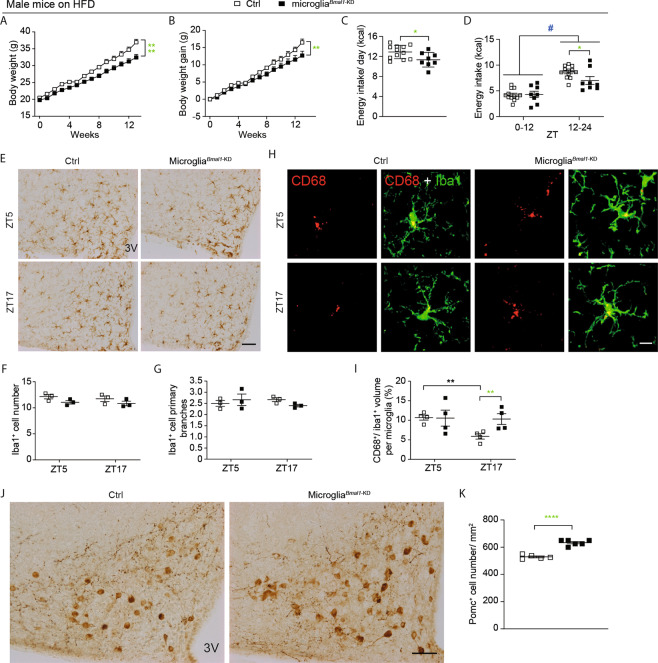
Functional and cellular impact of microglial Bmal1 deletion on HFD-induced obesity in male mice. **A** Absolute body weight and **B** body weight gain of Ctrl and microglia^*Bmal1*-KD^ mice fed a HFD (*n* = 5–7 mice per group). **C** Energy intake per day and **D** energy intake during light (ZT0–12) and dark phases (ZT12–24; *n* = 8–12 mice per group). **E** Representative images of Iba1 immunostaining in the ARC; 3 V, third ventricle. Scale bar, 100 µm. **F** Quantification of Iba1^+^ microglial cells and **G** Iba1^+^ primary branches (*n* = 2–3 mice per group). **H** Confocal images of CD68 and Iba1 immunostaining in the ARC; scale bar, 10 µm. **I** Quantitative analysis of the volume percentage associated with CD68^+^ microglia in the ARC (*n* = 4–5 mice per group; 100–125 cells were analyzed in each group). **J** Images of POMC^+^ neurons in the ARC; 3 V, third ventricle, scale bar, 100 µm. **K** Analysis of POMC^+^ neurons expression (*n* = 5–6 mice per group). Data are presented as means ± s.e.m. Green-colored * indicates a genotype effect. *P < 0.05, **P < 0.01, ****P < 0.0001, and ^#^*P* < 0.05 for ZT0–12 vs. ZT12–24.

Microglial activation in the hypothalamus has been observed in association with hypercaloric diet-induced obesity, a response that was shown to be sex-dependent [6, 29]. As such, we examined the metabolic phenotype of both male and female microglia^*Bmal1*-KD^ and Ctrl mice provided with either a standard chow diet or with a HFD. On the standard chow diet, no differences were observed with respect to any of the metabolic parameters evaluated in comparisons between microglia^*Bmal1*-KD^ and Ctrl, male or female mice (Fig. S2). Strikingly, we found that microglia^*Bmal1*-KD^ mice on a HFD gained significantly less weight than did their Ctrl counterparts (Fig. 2A, B). Differential weight gain was more prominent among females than males (Fig. S3A, B). Interestingly, microglia^*Bmal1*-KD^ male mice responded to the HFD with reduced food intake when compared to Ctrls (Fig. 2C); this response was observed mainly restricted to the dark phase in both sexes (Fig. 2D; Fig. S3C, D). Results from indirect calorimetry revealed that the mean RER increased in male Ctrls on the HFD, specifically during the dark, relative to the light phase, while no similar change was observed among microglia^*Bmal1*-KD^ mice (Fig. S3E, F). Mean RER increased during the dark phase among female microglia^*Bmal1*-KD^ mice relative to Ctrls (Fig. S3G, H). However, there was no genotype difference in daily physical activity and heat production in males (Fig. S3I–L) nor in females (Fig. S3M–P). Taken together, these data indicate that the microglia-specific *Bmal1* knock-down protects mice from HFD-induced obesity, mostly by lowering energy intake without affecting many other global metabolic parameters (RER, physical activity, heat production).

### Bmal1-deficiency results in increased microglial phagocytic capacity in response to HFD-induced metabolic challenge

The arcuate nucleus (ARC) of the hypothalamus plays a critical role in regulating systemic energy homeostasis [30]. We hypothesized that disruption of the circadian rhythm secondary to Bmal1-deficiency may have an impact on systemic homeostasis via modulation of microglial phagocytic capacity. Notably, our previous results obtained from experiments using a microglial cell line (BV2 cells) showed that deletion of Bmal1 by siRNA led to increased phagocytosis of microspheres compared to control cells [27]. In the current experiment, a similarly increased phagocytosis was observed in primary microglial cells isolated from microglia^*Bmal1*-KD^ mice (Fig. S4A, B). Subsequently, we assessed phagocytic capacity of microglia specifically in the ARC of both males and females after a 4-week trial of standard chow or HFD. Phagocytic capacity was evaluated at two specific time-points, ZT5 and ZT17, within a single 24-h period. We evaluated several parameters associated with overall phagocytic capacity, including the number and morphology of microglia identified by their characteristic Iba1 immunoreactivity (Iba1^+^ cells), coupled with co-immunostaining with the phagosome marker, CD68. Reduced levels of primary branching have been associated with high levels of phagocytic activity [23]. Microglial phagocytic activity can also be documented by the ratio of phagosome to soma volume (i.e., CD68^+^/Iba1^+^ ratio). Among our findings, we detected no differences in the number of Iba1^+^ cells or the number of primary projections when comparing tissue sections from microglia^*Bmal1*-KD^ and Ctrl, male and female mice, regardless of the dietary regimen (Fig. 2E–G, and Fig. S4C–K). Interestingly, the CD68^+^/Iba1^+^ ratio varied in a time-dependent manner in male Ctrl mice, with a significant decrease observed at ZT17 when compared to ZT5 (Fig. 2H, I). This decrease was not detected in tissue sections from microglia^*Bmal1*-KD^ mice, as the CD68^+^/Iba1^+^ ratio remained high at both time points. Indeed, there was a significant increase in the CD68^+^/Iba1^+^ ratio in microglia^*Bmal1*-KD^ mice at ZT17 compared to results from Ctrl mice at this time point (Fig. 2H, I). Although no specific time-dependent regulation was observed in Ctrl female mice, a significantly higher CD68^+^/Iba1^+^ ratio was detected in tissue sections from microglia^*Bmal1*-KD^ female mice compared to Ctrls at ZT5 and to microglia^*Bmal1*-KD^ mice at ZT17 (Fig. S5A, B). Of note, the CD68^+^/Iba1^+^ ratio remained unchanged in all mice provided with a standard chow diet (Fig. S5E, F, I, J). Taken together, these results suggest that the observed increase in CD68^+^ phagosomes within the Iba1^+^ microglia of microglia^*Bmal1*-KD^ mice may represent a specific response to metabolic stress (e.g., the HFD). It is also critical to note that we detected no significant changes in phagosome or soma volume (Fig. S5C, D, G, H, K–N). The results obtained from microglia^*Bmal1*-KD^ mice under HFD conditions are summarized in Table S1 and indicate that although the timing is different in male and female mice, the Bmal1-deficient microglia from both genders respond to the HFD with increased phagocytic capacity and thereby can affect more efficient clearance of the local microenvironment.

### Increased microglial phagocytic capacity may prevent decrease of POMC-immunoreactive neurons

Results from a previous study revealed that consumption of a HFD resulted in increased apoptosis of hypothalamic neurons [31]. Therefore, we hypothesized that the increased phagocytic capacity observed in microglia from microglia^*Bmal1*-KD^ mice may promote clearance of cellular debris, including DNA. To explore this issue, we performed a quantitative analysis of CD68^+^ phagosomes within Iba1^+^ microglial cells co-stained with 4′,6-diamidino-2-phenylindole (DAPI) (Fig. S6A, B). However, we found no differences in the ratio of CD68^+^ phagosomes that contained DNA fragments when comparing results from microglia^*Bmal1*-KD^ and Ctrl mice of both sexes (Fig. S6C, D). Interestingly, CD68^+^ phagosomes containing DAPI-stained DNA fragments were more prevalent in tissue sections from female mice (Fig. S6E). These results suggest that, when maintained on a HFD, female brain tissue was subject to higher levels of cellular apoptosis and/or has more effective global microglial phagocytic capacity than that in males.

The ARC includes a key population of anorexigenic POMC neurons that control food intake and body weight [32]. Defects of POMC expression cause severe obesity [33]. Additionally, HFD has been shown to induce the loss of POMC^+^ neurons [34, 35]. Therefore, we investigated whether the disrupted microglial circadian rhythms and increased phagocytic activity would have a specific impact on POMC^+^ neurons. We found that male microglia^*Bmal1*-KD^ mice on a HFD had more POMC^+^ neurons than did Ctrl mice on a HFD (Fig. 2J, K). A similar result was obtained in female mice (Fig. S7A, B). The larger number of POMC^+^ neurons in the ARC of microglia^*Bmal1*-KD^ mice on the HFD may contribute to diminished food intake and reduced body weight gain observed in these mice.

### Microglia-specific deletion of Bmal1 results in improved long-term memory consolidation and retention

**Fig. 3:**
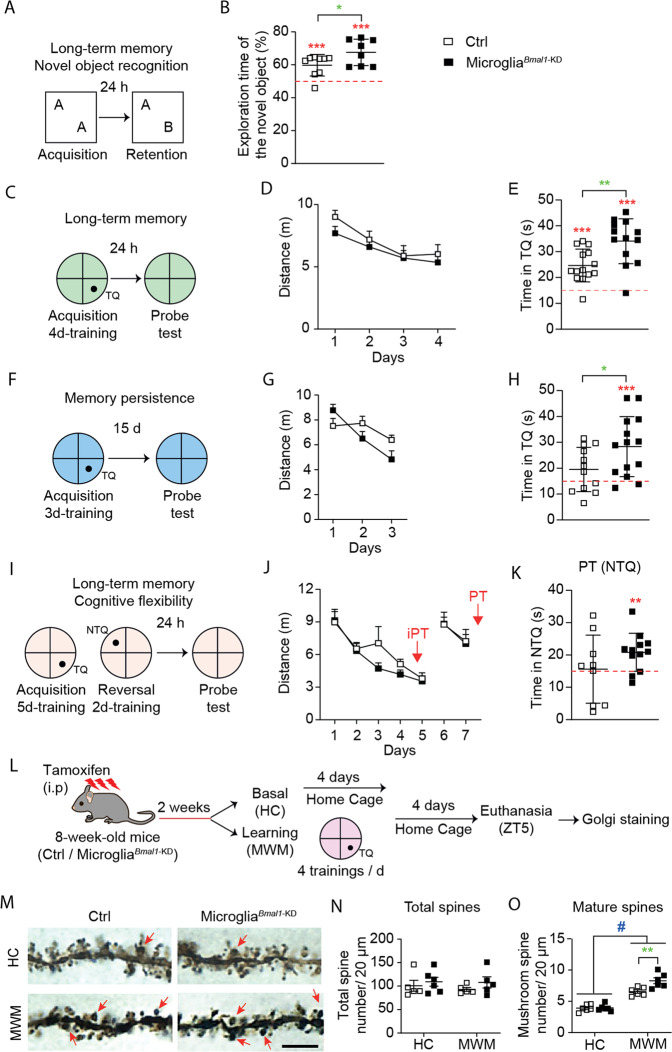
Microglia^*Bmal1*-KD^ mice exhibit improved long-term memory and more cognitive flexibility than Ctrl mice. **A** Experimental protocol and **B** performance in the novel object recognition (NOR) test. Included were microglia^*Bmal1*-KD^ (*n* = 8) and Ctrl (*n* = 9) mice. **C** Experimental protocol used to document long-term memory in the Morris Water Maze (MWM); TQ, Target Quadrant. **D** Distance required to reach the platform during each of the 4 days of acquisition training in the MWM. **E** Performance in the TQ during the probe test of microglia^*Bmal1*-KD^ (*n* = 13) and Ctrl (*n* = 14) mice. **F** Experimental protocol used to document memory persistence. **G** Distance required to reach the platform during the 3 days of limited training. **H** Performance in the TQ during the probe test of microglia^*Bmal1*-KD^ (*n* = 14) and Ctrl (*n* = 12) mice. **I** Experimental protocol used to document cognitive flexibility (reversal learning); NTQ, Novel Target Quadrant. **J** Distance required to reach the platform during the training. **K** Performance in NTQ during the probe test (9 Ctrl mice and 12 microglia^*Bmal1*-KD^ mice). **L** Experimental protocol prior to Golgi staining. **M** Images of dendritic spines of hippocampal pyramidal neurons. Arrow, mushroom spines; scale bar, 5 µm. Quantification of (**N**) total spines and (**O**) mature spines per 20 µm segment (*n* = 5–6 mice per group). Total spines were evaluated in 24 segments from four cells per mouse; mature mushroom spines were evaluated in 48 segments from six cells per mouse. Data are presented as means ± s.e.m. Green-colored * indicates a genotype effect; red-colored asterisk indicates an effect vs. random (15 s, red line); ^#^ when home cage (HC) are compared to MWM-trained mice; *P < 0.05, **P < 0.01, ***P < 0.001, and ^#^*P* < 0.05.

Microglia have the capacity to prune neuronal synapses, thereby establishing a mature connectivity pattern in the developing cortex and also in the adult hippocampus [36, 37]. As such, we considered the possibility that microglia^*Bmal1*-KD^ mice might experience different learning and memory behaviors when compared to Ctrls. We hypothesized that these differences might be most notable in experiments focusing on learning mechanisms that require synaptic rearrangements, such as memory consolidation [38]. We evaluated hippocampus-dependent long-term memory formation and consolidation in microglia^*Bmal1*-KD^ mice and Ctrls using several different protocols. It has been reported that the estrous cycle in female mice inevitably interferes with the spatial reference memory [39, 40], thus in the hippocampus-related experiments we only evaluated male mice. First, we evaluated long-term memory using the NOR test. This test is based on the observation that rodents display a spontaneous preference for novelty and are likewise capable of recalling previously encountered objects. The mice in this study were provided with 10 min to explore two identical objects placed in the center of an open field. Recognition memory was tested one day later; specifically, mice were exposed to one familiar object (the same one as encountered on the previous day) and one novel object (Fig. 3A). Intriguingly, we found that the microglia^*Bmal1*-KD^ mice spent more time interacting with the novel object than did the Ctrl mice. These results suggest that the microglia^*Bmal1*-KD^ mice are capable of significantly stronger long-term retention of a familiar object (Fig. 3B). We then tested the mice in the MWM, which is one of the most commonly used behavioral tests to test hippocampo-dependent memory. The MWM is used to evaluate different forms of spatial learning and memory in rodents, including consolidation, persistence, and reversal learning upon challenge with different protocols. For the MWM test, animals are placed in a pool filled with opaque water, and are provided with the opportunity to swim to a hidden escape platform placed in a target quadrant (TQ) which is located by relying upon outside environmental cues. After several days of acquisition training, the animals become more familiar with the task and the cues, and are thus able to locate the platform more quickly. A probe test is performed after completion of acquisition training. In this phase of testing, there is no platform in the pool; the time spent in the TQ provides the experimenter with an estimate of how effectively a given mouse can recall the original platform location. The first group of microglia^*Bmal1*-KD^ and Ctrl mice were subjected to 4-days of acquisition training with a fixed platform location. This was followed by a probe test performed 24 h later which was designed as described to assess long-term retention (Fig. 3C). No genotype-dependent differences were detected during the acquisition phase (Fig. 3D). Likewise, no differences between the mouse strains were detected with respect to measurements of latency (time required to locate the platform), swimming speed, or thigmotaxy (defined here as the time spent swimming along the border of the pool; Fig. S8A). Surprisingly, microglia^*Bmal1*-KD^ mice revealed higher performance on the memory retention (probe) test than was observed among the Ctrl mice (Fig. 3E). These results suggest that microglia^*Bmal1*-KD^ mice are capable of stronger long-term memory formation. The annulus crossing index, which represents the number of times the mice crossed over the platform in the TQ, was also superior in microglia^*Bmal1*-KD^ mice compared to Ctrls (Fig. S8A). These results indicated that the microglia^*Bmal1*-KD^ mice located the platform with a greater degree of precision.

A second group of mice was used to determine whether microglia-specific *Bmal1* knock-down would augment the memory consolidation process in mice subjected to a shorter or mild period of acquisition training. For these experiments, mice were provided with only 3 days of MWM acquisition training and were tested for memory retention at 15 days after the final training period (Fig. 3F). Under these conditions, Ctrl mice were unable to recall the correct location of the platform; this finding has been attributed to the limited extent of training associated with this protocol (Fig. 3H). However, microglia^*Bmal1*-KD^ mice were able to locate the platform in the TQ more readily than the corresponding Ctrl mice provided with the same limited period of acquisition training (Fig. 3H). Microglia^*Bmal1*-KD^ mice trained in this fashion also exhibited increased precision as demonstrated by the annulus crossing index (Fig. S8B). Distance, swimming speed, and thigmotaxy were not significantly different when comparing results from the two genotypes (Fig. 3G and Fig. S8B). Microglia^*Bmal1*-KD^ mice displayed increased acquisition performance relative to Ctrl mice with respect to latencies measured at day 3 of training (Fig. S8B).

### Microglia^*Bmal1*-KD^ mice exhibit improved cognitive flexibility

As our initial results suggested improved memory performance as measured in several experimental conditions, we next determined whether microglia^*Bmal1*-KD^ mice also exhibited increased perseverance or if they retained significant memory flexibility. These parameters were evaluated using a spatial reversal learning protocol. Mice were fully trained (5 days) in the MWM during which time each mouse memorized the fixed location of the platform in a TQ. The platform location was then changed to a new target quadrant (NTQ) within the same environment. Mice were then trained for an additional 2 days, and the time required to switch the new search strategy to locate the NTQ was determined. A probe test was performed 1 day later (Fig. 3I). We observed no significant differences with respect to distances traveled (Fig. 3J) or latencies associated with the platform location (Fig. S8C), regardless of its placement in the TQ or the NTQ. The microglia^*Bmal1*-KD^ mice showed less thigmotaxy than did the Ctrl mice, a finding that may indicate reduced anxiety (Fig. S8C). We confirmed that both mouse strains displayed significant retention with respect to the TQ, with microglia^*Bmal1*-KD^ mice exhibiting more precision during the intermediate probe trial (Fig. S8C). Importantly, in the reversal test, the microglia^*Bmal1*-KD^ mice spent significantly more time in the NTQ than would be expected based on random events; this was not observed among the Ctrl mice (Fig. 3K). Further evaluation of specific strategies revealed that microglia^*Bmal1*-KD^ mice had already initiated a direct swimming approach to the NTQ during the final 3 trials carried out on day 1; likewise, their actions revealed no perseverance or persistent focus on the earlier TQ during the final 3 trials carried out on day 2. By contrast, Ctrl mice exhibited a somewhat less direct swimming strategy on day 1 and residual perseverance toward the original TQ at the final trials carried out on day 2 (Fig. S8C). As such, our findings indicate that microglia^*Bmal1*-KD^ mice exhibit improved cognitive flexibility, as they were capable of more effective adaptation to new situations and challenges. Taken together, our results indicate that microglia-specific knockdown of *Bmal1* results in significant improvements in learning and memory processes in adult mice.

Structural plasticity, represented by the dynamic formation of dendritic spines and their stabilization over time in response to learning, is an important process associated with memory consolidation [41]. As such, we performed a quantitative assessment of the different forms of dendritic spines in the CA1 pyramidal neurons of the hippocampus under basal conditions and during the consolidation processes associated with spatial memory (Fig. 3L). We used the Golgi staining method to identify dendritic spines; typical results are as shown in Fig. 3M. We detected no differences with respect to total spine numbers when comparing results from microglia^*Bmal1*-KD^ mice to Ctrls. Specifically, we observed no differences between mice maintained in their home cages (HC; basal) and those subjected to the learning protocols (MWM; Fig. 3N). As previously observed in response to the MWM [42], we detected significantly more mushroom-type spines, which are structures that are formed and stabilized by learning processes, in both genotypes at day 4 after completion of the spatial learning process (Fig. 3O). However, microglia^*Bmal1*-KD^ mice displayed significantly larger numbers of mature spines per segment than did the Ctrl mice (Fig. 3O).

### Increased microglial phagocytic capacity detected in response to learning in the hippocampal stratum radiatum of microglia^*Bmal1*-KD^ mice

**Fig. 4:**
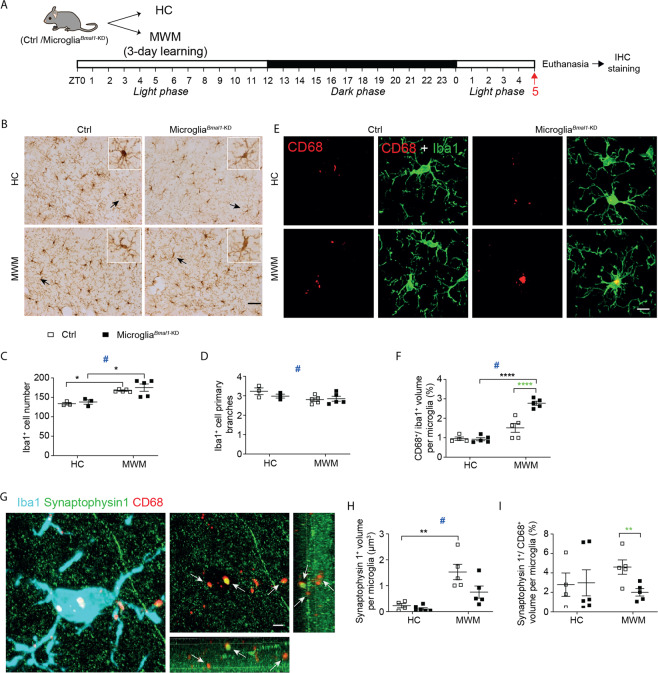
Phagocytic capacity of microglia in the hippocampal stratum radiatum is increased after learning. **A** Experimental strategy. Mice underwent 3 days of training in the MWM (4 trials/ day on days 1, 2, and 3) and were sacrificed at ZT5 on day 4. **B** Microglial immune reactivity in hippocampal CA1 performed with Iba1 immunostaining. Scale bar, 100 µm. Arrows indicate the Iba1-positive cell shown enlarged in the box (upper right corner). Quantification of **C** Iba1^+^ microglial cells and **D** primary branches in the hippocampal CA1 regions of mice in control group (HC; *n* = 3 mice per group) and those subjected to MWM training (*n* = 4–5 mice per group). **E** Confocal images documenting anti-CD68 and anti-Iba1 immunostaining in the hippocampal stratum radiatum; scale bar, 10 µm. **F** Quantitative analysis of the volume percentage of CD68^+^ microglia identified in the hippocampal stratum radiatum (HC, *n* = 4–5 mice per group; MWM, *n* = 5 mice per group; 100–125 cells were analyzed in each group). **G** Images documenting anti-Iba1, anti-synaptophysin 1, and anti-CD68 immunostaining within the hippocampal stratum radiatum. Arrows depict synaptophysin 1 and CD68 co-labeled phagosomes; scale bar, 3 µm. **H** Quantification of the volume represented by synaptophysin 1 in the CD68-positive phagosomes and **I** the ratio of synaptophysin 1^+^/CD68^+^ volume per microglial cell in hippocampal stratum radiatum (HC, *n* = 4–6 mice per group; MWM, *n* = 5 mice per group). Data are presented as means ± s.e.m. Green-colored * indicates a genotype effect; ^#^ control mice (HC) vs. those subjected to MWM training. *P < 0.05, **P < 0.01, ****P < 0.0001, and ^#^*P* < 0.05.

The findings from our study of microglia in the ARC suggested that their phagocytic capacity might increase in microglia^*Bmal1*-KD^ mice in response to a functional brain challenge. As such, we next analyzed microglial number and morphology, together with the phagocytic marker, CD68, in different regions of the hippocampus in mice that underwent MWM training (MWM) and those that did not (HC). Hippocampal regions, including the stratum oriens, the stratum radiatum, and the lacunosum moleculare (CA1 region), as well as the dentate gyrus were examined at 12 h after completion of the training period; this is represented as ZT17 on the same day, during the active phase (i.e., lights off; Fig. S9 and Fig. S10). We also examined responses at a 24 h after training, which is ZT5 during the day to follow, during sleeping phase, i.e., lights on; Fig. 4A and Fig. S10). Interestingly, MWM training resulted in an increase in microglial cell number and a decrease in microglial primary branches in both microglia^*Bmal1*-KD^ and Ctrl mice compared with the mice at baseline (HC) at both ZT5 (Fig. 4B–D) and ZT17 (Fig. S9B–D). This pattern was observed in all CA1 sub-regions, but not in the dentate gyrus (Fig. S10B–I). Taken together, these results suggest that MWM training has a profound influence on microglial cells in the hippocampus. Remarkably, MWM training also induced a significant increase in CD68^+^/Iba1^+^ volume ratio in both genotypes compared to findings from mice at baseline (HC). This response was notably higher in the microglia^*Bmal1*-KD^ mice compared to Ctrls at ZT5 (Fig. 4E, F), but not at ZT17 (Fig. S10E, F), again suggesting that microglia may respond to spatial learning with an increase in microglial phagocytic capacity. The increased CD68^+^/Iba1^+^ volume ratio most likely resulted from an increase in CD68 immunoreactivity that developed in response to MWM training with Iba1^+^ microglial soma volumes remaining unchanged (Fig. S10J, K). Intriguingly, these responses were not observed in either the hippocampal stratum oriens or in the lacunosum moleculare (Fig. S10L–S). These results indicate that microglia-mediated clearance activities may vary in different regions of the brain and also point to the apical dendrites of hippocampal CA1 pyramidal neurons as the main microglia targets associated with spatial learning. Together, these data indicate that MWM training promotes microglial phagocytic activity in the stratum radiatum of both Ctrl and microglia^*Bmal1*-KD^ mice, and that this response is more pronounced in the latter strain.

Given that the role of microglia in remodeling synapses involves phagocytosis of presynaptic structures, we performed experiments designed to assess levels of synaptophysin 1 within CD68^+^ microglial phagosomes. First, we found that MWM training had a global and significant impact on synaptophysin 1 levels, which were increased over baseline in both microglia^*Bmal1*-KD^ and Ctrl mice at both ZTs (ZT5, Fig. 4G, H, and ZT17, Fig. S9G). Second, our evaluation of synaptophysin 1 content in microglia, presented as the synaptophysin 1^+^/CD68^+^ volume ratio, revealed no differences among mice in the basal (HC) group. However, this ratio was significantly diminished in microglia from microglia^*Bmal1*-KD^ mice compared to Ctrls at ZT5 after MWM training (Fig. 4I) while they did not differ at ZT17 (Fig. S9H). Taken together, these data indicate that while MWM training induced phagocytotic activity in microglia at ZT5, the presynaptic marker synaptophysin 1 was more prominently phagocytosed by the Ctrl than by the *Bmal1*-KD microglia at that time point.

## Discussion

Microglia are brain macrophages that play important roles in regulating systemic energy homeostasis and cognition in rodent species. Microglial immune activity follows a circadian rhythm [7]. As such, our study focused on the intrinsic circadian clock of microglia. Specifically, our experiments were designed to determine how cell-specific disruption of microglial circadian rhythmicity affects systemic energy homeostasis, learning, and memory processes in mice. Our findings demonstrate that microglia-specific knock-down of the clock gene *Bmal1*, promotes microglial phagocytosis in the hypothalamus in mice responding to HFD-induced metabolic stress and likewise in the hippocampus in response to learning and memory processes. As a result, mice with a microglia-specific Bmal1 deficiency exhibit decreased HFD-induced hyperphagia and body weight gain and also have an improved capacity for long-term memory consolidation and retention, while retaining cognitive flexibility.

First, we observed enhanced microglial phagocytosis in the ARC in association with a reduced loss of POMC-immunoreactivity and resistance to gain weight in response to HFD in both male and female microglia Bmal1-KD mice. Growing evidence from both human and rodent studies has revealed that HFD-induced obesity is associated with hypothalamic gliosis and microglial inflammation [43]. However, the role played by the microglial phagocytosis in this process has not been fully clarified. We recently reported that downregulation of microglial phagocytic capacity exerts a detrimental effect on the survival and function of surrounding POMC^+^ neurons in the setting of a hypercaloric environment [6]. Our current data reveal that enhanced microglial phagocytic capacity may serve to prevent the reduction of POMC^+^ neurons from the negative sequelae associated with a HFD. Interestingly, activation of POMC neurons results in decreased food intake and less body weight gain [32]. As such, our findings suggest that the circadian rhythm within the hypothalamic microglia has a direct impact on its phagocytosis capacity and plays a critical role in regulating hypothalamic neural control of body weight.

Second, we observed enhanced microglial phagocytosis in the hippocampus in association with increased formation of mature dendritic spines and enhanced memory processes. In order to form long-term memory, synapses from engram cells are strengthened and new spines support additional interconnections between these cells. These two processes are believed to participate in memory storage [44, 45]. New stable dendritic spines of the mushroom-type are indeed produced in CA1 of rodents upon spatial learning in the MWM [41, 42]. The consolidated engram may last for some time and, as circuits rewiring occurs in response to new experience and integration of newborn neurons (adult neurogenesis) [46], the potentiated synapses may weaken over retention time leading to forgetting. Interestingly, it has recently been suggested that the synaptic elimination by microglial phagocytosis may result in normal forgetting [47]. It seems that weak synapses are tagged for removal in the adult network, a process previously demonstrated to occur during development to strengthen networks [4]. Our results showing increased memory consolidation, retention, and flexibility in microglial Bmal1-KD mice are suggesting that microglial engulfing of weak synapses may be more efficient when microglial phagocytotic activity is acting “on-demand” rather than restricted by the circadian clock. Further, microglia were recently shown to have a facilitating role in synaptic circuit remodeling and maturation as they exert selective partial phagocytosis of presynaptic structures and could induce postsynaptic spine head filopodia [37]. Thus, our study evidencing that the presynaptic marker synaptophysin 1 is less engulfed into phagosomes of microglia^*Bmal1*-KD^ mice than Ctrls 24 h after the series of learning trials, suggests that Bmal1-KD microglia may better help in postsynaptic spine consolidation. This is emphasized by our result showing a significant increase of mushroom-type spines after learning in microglia^*Bmal1*-KD^ mice. Yet, *Bmal1*-KD microglia may additionally better clean the environment of cellular debris and prevent inflammatory events to occur. Appropriate microglial phagocytic capacity is crucial for effective synaptic pruning and likewise for the formation of mature neural circuits.

Our results showed some sex differences in Bmal1-KD microglial clock gene expression. It is well known that the circadian system differs between the sexes and circadian clocks may be modulated by estrogen receptor signaling [48–50]. The influence of gonadal hormones on the circadian system may also result in the differential expression of clock genes in male and female Bmal1-KD microglia, but for now, the mechanism remains unknown. Moreover, recently it has been reported that in male rats the synaptic phagocytosis of microglia follows a circadian rhythm under physiological conditions [51]. Here we observed that in both genders Bmal1-KD microglia exhibit increased phagocytosis, indicated by CD68 immunoreactivity, during HFD feeding, but with different timing for males and females. This difference may be caused by the sex different clock gene expression in Bmal1-KD microglia. We further explored phagocytosis of DNA debris in hypothalamic microglia after several weeks on a HFD. As shown, no significant differences were found when comparing Bmal1-deficient microglia to those from Ctrl mice. However, we did detect more DNA fragment-containing phagosomes in microglia from females, as opposed to male mice. This finding suggests that HFD-induced neuronal loss may be sex-dependent.

However, microglial phagocytic capacity also depends on specific neuronal activity and the rate of neuronal attrition [23]. Neural circuits involved in responses to challenges such as HFD-induced metabolic stress or learning tasks require microglial phagocytosis “on-demand”. As such, suppression of the rigorous control provided by the circadian clock may have a beneficial impact on microglial function. In this study, microglia-specific knock-down of Bmal1 promoted increased microglial phagocytosis likely producing a healthier microenvironment for neighboring neurons in the hypothalamus and hippocampus. Deficiency of microglial Bmal1 ultimately protected mice from HFD-induced obesity and increased memory performance. Interestingly, the beneficial impact of this loss-of-function perfectly fits with recent findings reported for global Bmal1 knockout mice, in which both locomotor activity and insulin sensitivity were found to adapt more readily to disrupted light/dark schedules compared to the responses of these pathways in *Bmal1*-sufficient controls [52]. Furthermore, in the respiratory tract, *Bmal1*-deficiency has been associated with increased phagocytic function and enhanced macrophage-mediated antibacterial immunity [53]. Clearly, when behavioral rhythms are disrupted it may be beneficial for optimal health that microglial activity is controlled less strictly by its internal clock.

Microglia express both Bmal1 and pro-inflammatory cytokines at comparatively high levels during the light phase [7]. Of note, previously we showed that microglia-specific *Bmal1* deficiency reduced inflammation-related gene expression in vivo [27], whereas LPS-induced IL6 up-regulation was found reduced in absence of microglial Bmal1 [22]. Activated microglia undergo polarization toward pro-inflammatory or anti-inflammatory phenotypes [54]. As such, our findings, including decreased inflammation [27] and increased phagocytic capacity, suggest that Bmal1-deficient microglia tend to become polarized toward an anti-inflammatory state. Future work will be required to identify mechanisms linking Bmal1 and microglia-mediated phagocytosis.

Microglial phagocytosis differs in distinct regions of the brain [23]. However, in microglia^*Bmal1*-KD^ mice, a circadian rhythm persisted as food intake, RER, locomotor activity, and heat production were not globally changed. This suggests that the microglia-specific deficiency of Bmal1 does not have a direct impact on the central pacemaker in the suprachiasmatic nucleus. We conclude that phagocytosis increases in Bmal1-deficient microglia localized specifically within the hypothalamic ARC and hippocampal stratum radiatum as an “on-demand” response to external challenges such as HFD-induced metabolic stress and learning trainings, respectively. The observed increase in phagocytosis may have a beneficial impact on microglial control of energy balance and cognition, which is reminiscent of psychiatric treatment. Together, these findings indicate that agents that target the molecular clock-Bmal1 in microglial cells might be developed as a novel means to treat both metabolic and cognitive disorders.

## Supplementary information

Supplemental information

## Data Availability

The authors confirm that the data supporting the findings of this study are available within the article and the Supplementary Material. Additional data related to this paper may be requested from the authors.
