# Supplementary material for: Microglia-specific knock-down of Bmal1 improves memory and protects mice from high fat diet-induced obesity
Source: Mol Psychiatry. 2021 May 28;26(11):6336–49. doi: 10.1038/s41380-021-01169-z (PMC8760060; doi:10.1038/s41380-021-01169-z)
Supplement: Supplementary file 1 — Supplemental information [file 41380_2021_1169_MOESM1_ESM.docx]

**Supplemental Information**

**Microglia-specific knock-down of Bmal1 improves memory** **and** **protects mice from** **high fat** **diet-induced obesity**

Xiao-Lan Wang^1,2,3^, Sander Kooijman^4^, Yuanqing Gao^2,3^, Laura Tzeplaeff^1^, Brigitte Cosquer^1,5^, Irina Milanova^2,3^, Samantha E.C. Wolff^3^, Nikita Korpel^2,3,6^, Marie-France Champy^7,8^, Benoit Petit-Demoulière^7,8^, Isabelle Goncalves Da Cruz ^7,8^, Tania Sorg-Guss^7,8^, Patrick C.N. Rensen^4^, Jean-Christophe Cassel^1,5^, Andries Kalsbeek^2,3,6^, Anne-Laurence Boutillier^1,5^ *, Chun-Xia Yi^2,3.6^ *

^1^Université de Strasbourg, Laboratoire de Neuroscience Cognitives et Adaptatives (LNCA), Strasbourg, France

^2^Department of Endocrinology and Metabolism, Amsterdam University Medical Centres (UMC), University of Amsterdam, Amsterdam, the Netherlands

^3^Laboratory of Endocrinology, Amsterdam University Medical Centres (UMC), University of Amsterdam, Amsterdam Gastroenterology & Metabolism, Amsterdam, the Netherlands

^4^Department of Medicine, Divison of Endocrinology, and Einthoven Laboratory for Experimental Vascular Medicine, Leiden University Medical Center, Leiden, the Netherlands

^5^CNRS UMR 7364, LNCA, Strasbourg, France

^6^Netherlands Institute for Neuroscience, an Institute of the Royal Netherlands Academy of Arts and Sciences, Amsterdam, the Netherlands

^7^PHENOMIN-ICS, Institut Clinique de la souris, CNRS, UMR7104, Illkirch, France

^8^INSERM, U964, Illkirch, France; Université de Strasbourg, France

*Equal contribution, joint last authors, and corresponding authors

* Co-corresponding and joint last author:

Anne-Laurence Boutillier Ph.D.

Laboratoire de Neurosciences Cognitives et Adaptatives (LNCA)

CNRS UMR 7364, Université de Strasbourg

12, rue Goethe 67000 Strasbourg, France
Phone: +33368851934

[laurette@unistra.fr](mailto:laurette@unistra.fr)

* Co-corresponding and joint last author:

Chun-Xia Yi M.D. Ph.D.

Department of Endocrinology and Metabolism

Amsterdam University Medical Centres (UMC)

Meibergdreef 9, 1105AZ, Amsterdam, The Netherlands

Phone: +31205664807

Fax: +31206917682

[c.yi@amsterdamumc.nl](mailto:c.yi@amsterdamumc.nl)

**Supplemental Materials and Methods**

**Animals**

Both microglia*^Bmal1^*^-KD^ and Ctrl mice were injected with tamoxifen as noted above. Genotyping of *Bmal1*^lox/lox^ and *Cx3cr1*^CreER^ was performed by polymerase chain reaction (PCR) using the following primers: *Bmal1*^lox/lox^ primers, 5’– ACT GGA AGT AAC TTT ATC AAA CTG – 3’ and 5’– CTG ACC AAC TTG CTA ACA ATT A – 3’; *Cx3cr1*^CreER^ primers, 5’– AAG ACT CAC GTG GAC CTG CT – 3’, 5’– CGG TTA TTC AAC TTG CAC CA – 3’, and 5’ – AGG ATG TTG ACT TCC GAG TTG – 3’. Mice were housed in a temperature (22 ± 1°C) and humidity (55 ± 5%) controlled room under a 12 h light/dark cycle (lights on at 07:00 h, Zeitgeber Time 0 [ZT0]), with free access to food and water. Mice were fed a standard chow (Mucedola Srl, Settimo Milanese, Italy, cat. no. 4RF21). Both male and female mice were used for the metabolic phenotype study, while only male mice were used in the Morris Water Maze (MWM) and New Object Recognition (NOR) protocols. All tests are routinely used in our laboratory and sample size was decided based on our previous studies ([1](#_ENREF_1),[2](#_ENREF_2)). Animals were randomly assigned to experimental groups. Experimental and animal care protocols were in compliance with the institutional guidelines (national and international laws and policies: directive 2010/63/UE, February 13, 2013, European Community). Our project has been reviewed and approved by the national and regional ethics committee, France (APAFIS#6822-2016092118336690v3 and APAFIS#9631-201801251042855 v1). For Figure 1 microglia were isolated from 10-week-old male wild-type (C57BL/6J) mice that were killed every 3 h beginning at ZT0. This study was approved by and performed according to the guidelines of the Institutional Animal Care and Use Committee of the Netherlands (Leiden, The Netherlands).

**Indirect calorimetry**

### Mice were housed individually in TSE cages for 48 hrs; this period included a 24 hrs period for habituation and another 24 hrs for the measurement of energy expenditure. Oxygen consumption (VO_2_) and carbon dioxide production (VCO_2_) were measured every hour during this 24 hrs period to calculate RER using the formula RER = VCO_2_/VO_2_, which defines fuel preference (glucose *vs.* lipid metabolism). Heat production was calculated using the formula Heat = [3.941(VO_2_) + 1.106(VCO_2_)] x 1.44. Physical activity, which is a component of whole energy expenditure, was also recorded. These experiments were performed at the French National Infrastructure for Mouse Phenogenomics (PHENOMIN) and received an ethical authorization by the French Ministry of Research, in compliance with the European Community regulation for laboratory animal care and use (Directive 2010/63/UE).

**Morris Water Maze (MWM) Task**

*Acquisition phase* Mice were placed in a circular pool (150 cm diameter) filled with opaque water at 20 ± 1°C in an experimental room that contained cues and paintings on the walls. The platform (10 cm diameter) was positioned in the southeast (SE) quadrant of the pool at 1 cm below the water surface. Mice were first subjected to a 1-day habituation trial with a visible platform; this was followed by 3, 4, or 5 days of acquisition training (4 trials/day) with a hidden platform. For each trial, the mouse was placed at the edge of the pool at a different, random starting position and provided with a maximum of 60 s to reach the submerged platform. Upon reaching the platform, the mouse was left in place for 10 s prior to initiating another round via placement at the next starting point. If a mouse failed to locate the platform within 60 s, it was gently guided to it by the experimenter and was permitted to remain in place for 10 s. A mouse would be excluded if it was floating more than 50% of the time in the pool.

*Probe trial.* All mice were tested for memory retention in the probe trial which was administered at 24 hrs and 15 days after the final acquisition trial as described above. For this test, the platform was removed, and all mice were released from the center of the pool. Responses were evaluated after 60 s of swimming.

*Reversal phase.* After 5 days of acquisition training (4 trials/day) with the platform in the SE position, mice were trained for another 2 days (4 trials/day) with the platform moved to the opposite (i.e., northwest [NW]) quadrant. During reversal training, the mouse was placed at the edge of the pool at different, random starting positions and provided with a maximum of 60 s to reach the submerged platform. Upon reaching the platform, the mouse was left in place for 10 s prior to initiating another round via placement at the next starting point. If a mouse failed to find the platform within 60 s, it was moved to its home cage by the experimenter. A probe trial as described above was performed on day 8 at 24 hrs after the final reversal training trial.

Parameters were recorded using a video-tracking system (ANY-maze, Ugo Basile, Italy). The experimenter was blinded with respect to mouse genotype. Swim path, swim speed, latency, and distance covered to reach the platform were measured during the acquisition trial. Time spent in each of the four quadrants and the number of platform crossings were recorded and calculated for the probe trial. We addressed learning behavior during acquisition together with the different retention times with respect to the MWM test as well as cognitive flexibility during reversal training.

**Novel Object Recognition (NOR) test**

Mice were habituated to a 52 x 52 x 52 cm box with three different components: familiarization, acquisition, and retention phases. On day 1, mice were subjected individually to a single 10-minute familiarization session. During this time, each mouse was introduced to the empty arena and was permitted to become familiar with the apparatus. On day 2, each mouse was subjected to a single 15-minute habituation session, during which time two same objects (A1 and A2) were placed in symmetrically with respect to the center of the arena. Mice were returned to their home cages for 24 hrs, and then re-introduced to the arena where they were exposed to one familiar (A) and one new object (B) that were placed at the same locations as were the sample stimuli. Mice were provided with a 10-minute period to explore the objects. Exploration was defined as sniffing or touching the objects with the nose and/or forepaws. The arena and all objects were cleaned with 70% ethanol between each session to ensure the absence of olfactory cues. All training and testing sessions were digitally recorded (TopScan, CleverSys, Inc., Reston, VA, USA) and analyzed by the experimenter who was blinded to the mouse genotype. Behavioral experiments were performed only once with each mouse. All experimental groups were fully independent of one another.

**Labeling and counting dendritic spines after Golgi staining**

Mice subjected to 4-day training in the MWM were sacrificed at day 4 after the final training period. The Rapid Golgi stain kit (FD Neurotechnologies, Inc., Columbia, MD, USA) was used according to the manufacturer's instructions. One hundred μm thick coronal sections containing the hippocampal region CA1 were cut using a Vibratome (VT1000M, Leica Biosystems, Buffalo Grove, IL, USA). Integrated neuronal fragments are chosen for spine counting and spines are analyzed manually using a bright-field microscope equipped with an automated motorized stage and MorphoStrider software (Explora Nova, La Rochelle, France). Thus, morphologies of spines are dynamically identified one by one, in regard to its shape throughout the z-axis, according to the size of the spine head and length of the spine neck. For each hemisphere, three neurons are chosen in the counting window. To-be-analyzed neurons are selected under the light-transmission microscope using low magnification. A neuron has to respond to three criteria to be selected for quantification, as in Restivo et al. ([2009](https://link.springer.com/article/10.1007/s00429-019-01865-1#ref-CR34)) ([3](#_ENREF_3)): 1) the dendrite had to be untruncated, 2) staining and impregnation had to be homogenous along with the entire extent of the dendrite, and 3) neurons had to be easily discernible and relatively well isolated from neighboring impregnated cells. Measurements are performed on apical and basal dendrites in each region, at least 50 µm away from the soma for the apical dendrites, and at least 30 µm away for the basal dendrites, on secondary and tertiary branches. These distances allow us to exclude dendritic segments near the soma that are essentially devoid of spines. For each neuron, 20-µm long segments are randomly selected on apical and basal dendrites within a distance of at most 100 µm from the limit of the exclusion zone. Counting is performed under a 1000 × magnification using an oil immersion objective. Spines are counted blind to the experimental conditions. For quantification of total spines, 24 dendritic segments (20 µm) were analyzed (4 cells per mouse, n = 6 mice per group). For quantification of mushroom spines, 48 dendritic segments were analyzed (6 cells per mouse, n = 6 mice per group).

**Isolation of microglia from brain tissue of adult mice**

Three weeks after the tamoxifen injections, mice were decapitated for extraction of brain tissue. Brain tissue was gently hand-homogenized in Roswell Park Memorial Institute (RPMI) 1640 medium (Gibco, Gaithersburg, MD, USA; cat. no. 11875093) and filtered through a 70 μm cell strainer (Corning, Inc., Corning, NY, USA; cat. no. 431751). After 5 min centrifugation at 380 x *g* at 4 °C, cells were re-suspended in 7 ml RPMI 1640 medium and mixed with 3 ml stock isotonic Percoll (SIP) solution (1 part 10X Hank’s buffered saline solution [10X HBSS; Gibco, cat. no. 14185052] with 9 parts Percoll plus [Sigma-Aldrich, St. Louis, MO, USA, cat no. GE17-5445-01]). The cell suspension was layered slowly on top of 2 ml of a 70% Percoll solution (3 parts of HBSS (Gibco, cat. no. 14170112) with 7 parts SIP in a fresh 15 ml Falcon tube and centrifuged at 500 x *g* for 30 min at 18 °C, with minimal acceleration and breaking. After centrifugation, material that collected in the interphase was transferred into a fresh 15 ml Falcon tube, diluted with 8 ml HBSS, and centrifuged at 500 x *g* for 7 min. The supernatant was then discarded and isolated microglia were incubated with anti-mouse CD11b antibody-conjugated magnetic microbeads (Miltenyi Biotec, Bergisch Gladbach, Germany, cat. no. 130-093-634) for 15 min at 4°C. The CD11b^+^ microglia were isolated using a MACS Column (Miltenyi Biotec, cat. no. 130-042-201) with an applied magnetic field. Microglial cells were collected for RNA isolation and protein extraction.

**RNA isolation from isolated microglia and quantitative reverse-transcription polymerase chain reaction (qPCR)**

Total RNA was isolated from freshly-isolated microglia using an RNeasy Micro Kit (QIAGEN, Hilden, Germany, cat. no. 74004) following the manufacturer’s recommendations. We used 150 ng of microglial RNA to generate cDNA with a Transcriptor First Strand cDNA Synthesis Kit (Roche, Basel, Switzerland, cat. no. 04897030001) following the manufacturer’s recommendations. Quantitative PCR (qPCR) was performed using a SensiFAST™ SYBR® No-ROX Kit (Roche Bioline, cat. no. BIO-98020). Data were analyzed by LC480 Conversion and LinRegPCR software with normalization to expression levels of the housekeeping gene, hypoxanthine phosphoribosyltransferase 1 (HPRT1).

**Western blot analyses**

Isolated microglial cells were homogenized in Laemmli buffer and sonicated twice for 10 s (ultrasonic processor, with power at 40%) followed by heating at 70°C for 10 min and then at 100°C for 5 min. Lysates were centrifuged at 14,000 x g for 5 min and supernatants were used for Western blot analyses. Proteins were loaded on Midi‐PROTEAN TGX Stain‐Free™ Precast Gels (4–20%, Bio‐Rad, Hercules, CA, USA) and electrotransferred onto a nitrocellulose membrane. Primary antibodies used to probe Western blots included rabbit anti-Bmal1 (1:500 dilution, Novus Biologicals, Littleton, CA, USA, cat. no. NB100-2288) and rabbit anti-histone H3 (1:1000 dilution, Abcam, Cambridge, UK, cat. no. ab1791). Horseradish peroxidase-conjugated anti-rabbit Ig (1:5000 dilution, Jackson ImmunoResearch, West Grove, PA, USA) was used as the secondary antibody. Immunoreactive bands were detected with ECL (Clarity, Bio‐Rad) using a ChemiDoc Touch system (Bio‐Rad). Results were quantified using ImageLab software.

**Primary microglial culture and phagocytosis assay**

Primary microglial cells were prepared as described previously ([4](#_ENREF_4)). Briefly, brain tissues were isolated from P1-P3 microglia*^Bmal1^*^-KD^ and littermate Ctrl mice, the meninges and blood vessels were removed, the parenchyma was cut into smaller pieces, and dissociated in Trypsin-EDTA solution (T4049, Sigma) at 37 °C for 8 min. Cell suspension went through 70 μm cell strainer (431751, Corning) twice and seeded in DMEM/F12 (10565018, Gibco), containing 10% FBS, 100 μg/ml penicillin-streptomycin, 5 ng/ml M-CSF (SRP3221, Sigma). Six to ten days later, the flasks were shaken (200 rpm) for 1 hr to release microglia and harvested microglia were cultured on coverslips in medium without M-CSF. Microglial cells isolated from microglia*^Bmal1^*^-KD^ mice were treated with 5 μM of 4-hydroxytamoxifen (SML1666-1ML, Sigma) for 48 hrs to induce Cre-LoxP recombination and excise Bmal1, while cells isolated from Ctrl mice were treated with same volume of vehicle (DMSO). Next, microglia were synchronized with 100 nM dexamethasone (D1756, Sigma) for 2 hrs, washed with PBS for further treatment.

Fluoresbrite® Polychromatic Red Microspheres (1.0 µm, 18660-5, polysciences) were coated with 10% FBS at 37 °C for 1 hr, followed by centrifugation (12,000 rpm, 2 min) and resuspension in PBS. Coated microspheres were added to the synchronized primary microglial cells (100 microspheres per cell), and 1 hr later, cells were washed with PBS 3 times, then fixed by 4% paraformaldehyde for 5 min, followed by PBS washing. Coverslips were incubated with guinea pig anti-Iba1 (1:500 dilution, Synaptic Systems, cat. no. 234004) for 2 hrs at room temperature, followed by incubation with Alexa Fluor 488-conjugated donkey anti-guinea pig Ig (1:400 dilution, Jackson ImmunoResearch, cat. no. 706546148) for 1 hr at room temperature. Imaging was captured using a fluorescence microscope (ApoTome.2, Zeiss, Germany) with a 20x objective at 1× zoom. Microspheres per cell were analyzed manually in Image J.

### Characterizing neurons and microglial cells in the hypothalamus and hippocampus

### Mice were anesthetized with pentobarbital (50 mg/kg) and subjected to a 2-min transcardial perfusion with phosphate buffered saline (PBS) followed by cold 4% paraformaldehyde (PFA) for 8 min. Brains were removed and post-fixed overnight in 4% PFA and stored for an additional 48 hrs at 4^o^C in a 30% sucrose solution for cryoprotection. Brains were cut into 30 μm thick coronal sections on a cryostat. Sections were stored in cryoprotectant solution at -20°C prior to immunostaining. For immunostaining, brain sections containing the dorsal hippocampus or mediobasal hypothalamus (MBH) were rinsed in tris-buffered saline (TBS) and incubated with primary antibodies overnight at 4°C. Primary antibodies used for immunostaining included rabbit anti-Iba1 (1:400 dilution, Synaptic Systems, Gottingen, Germany, cat. no. 234003), rat anti-CD68 (1:100 dilution, Abcam, cat. no. ab53444, guinea pig anti-synaptophysin 1 (1:400 dilution, Synaptic Systems, cat. no. 101004), and rabbit anti-POMC (1:1000 dilution, Phoenix Pharmaceuticals Inc., Burlingame, CA, USA, cat. no. H-029-30). Brain slices were then rinsed and incubated with secondary antibodies for 1 hr at room temperature. Secondary antibodies included biotinylated goat anti-rabbit Ig (Vector Laboratories, Inc., Burlingame, CA, USA, cat. no. BA-1000), biotinylated goat anti-rat Ig (Vector Laboratories, cat. no. BA-9400), Alexa Fluor 488-conjugated donkey anti-rabbit Ig (Invitrogen, Carlsbad, CA, USA, cat. no. A21206), Alexa Fluor 594-conjugated streptavidin (Jackson ImmunoResearch, cat. no. 016580084), and Alexa Fluor 647-conjugated donkey anti-guinea pig Ig (Jackson Immunoresearch, cat. no. 706605148), all diluted to 1:400. Samples were mounted with antifade mounting medium (Vector Laboratories, cat. no. H-1000) or antifade mounting medium with 4′,6-diamidino-2-phenylindole (DAPI, Vector Laboratories, cat. no. H-1200).

For immunohistochemical staining, sections were rinsed and incubated with biotinylated secondary antibody and avidin-biotin complex (ABC method, Vector Laboratories), followed by incubation in 1% diaminobenzidine with 0.01% hydrogen peroxide for 3 min. Sections were then rinsed with TBS and mounted on Superfrost Plus microscope slides (Fisher Scientific, Waltham, MA, USA). Sections dried thoroughly, followed by dehydration and delipidation via a series of ethanol washes and xylene, respectively. Imaging was performed using a brightfield microscope (Leica Biosystems, Heidelberg, Germany) with a 10x or 20x objective.

For immunofluorescence staining, fluorescent-conjugated antibodies were added as described. After several rinses, brain sections were mounted on Superfrost Plus microscope slides, covered with cover slips, and stored at 4°C. Imaging was performed using a confocal microscope (TCS-SP8, Leica Biosystems, Heidelberg, Germany). The laser power, photomultiplier gain, and offset were maintained constant for all images. For anti-Iba1 and anti-CD68 double staining with or without DAPI, 20 μm z-stack confocal images were acquired at 1 μm or 0.5 μm intervals, using a 40× /1.3 oil objective at 1× or ~4× zoom. For anti-Iba1, anti-synaptophysin 1, and anti-CD68 triple staining, 16 μm z-stack confocal images were acquired at 0.5 μm intervals, with 63× /1.4 oil objective, also at 1× or ~4× zoom.

**Imaging quantification**

Immunohistochemistry images were analyzed using Image J. Iba1^+^ cells and primary projections were counted manually in a 300 μm x 300 μm frame for quantification in the fixed region of ARC. POMC-immunoreactive (POMC^+^) cells were counted from every 5th section between Bregma -1.34 to -2.18 (Paxinos Mouse Brain Atlas) in each animal, i.e. about 8 sections/animal. MorphoStrider software (Explora Nova, La Rochelle, France) was used to analyze the surface area of ARC in each section and the POMC^+^ cell number was counted manually in the same region. Immunofluorescent images were analyzed using Imaris 8.3 software (Bitplane AG, Zurich, Switzerland); z-stack confocal images were constructed and photographed under a 40× /1.3 or 63× /1.4 oil objective at 1× zoom. The volume of CD68^+^ particles, Iba1^+^ microglial cells, synaptophysin 1, and DAPI^+^CD68^+^ microglia were determined for each brain section using Imaris 8.3 and presented as the average value per mouse.

**Supplemental Data**

**Supplementary Table 1. Summarized data obtained from both male and female mice (Bmal1-KD *versus* Ctrl).**

|  | Isolated microglia |  | HFD condition | | | | | | |
| --- | --- | --- | --- | --- | --- | --- | --- | --- | --- |
|  | Gene  expression |  | Body weight | Energy intake | RER | Physical activity | Heat production | Microglial  cell number | Phagocytosis |
| Male | Bmal1 🡫, Cry 🡩 |  | 🡫 | 🡫 | None | None | None | None | 🡩 (at ZT17) |
| Female | Bmal1 🡫, Dbp 🡫 |  | 🡫 | 🡫 (P=0.051) | 🡩 | None | None | None | 🡩 (at ZT5) |

Arrow (🡩 or 🡫) indicates a significant genotype difference (Bmal1-KD *versus* Ctrl); 🡩 means increased effect in Bmal1-KD group, while 🡫 means decreased effect. None indicates no genotype difference.

**Supplemental Figure. 1**


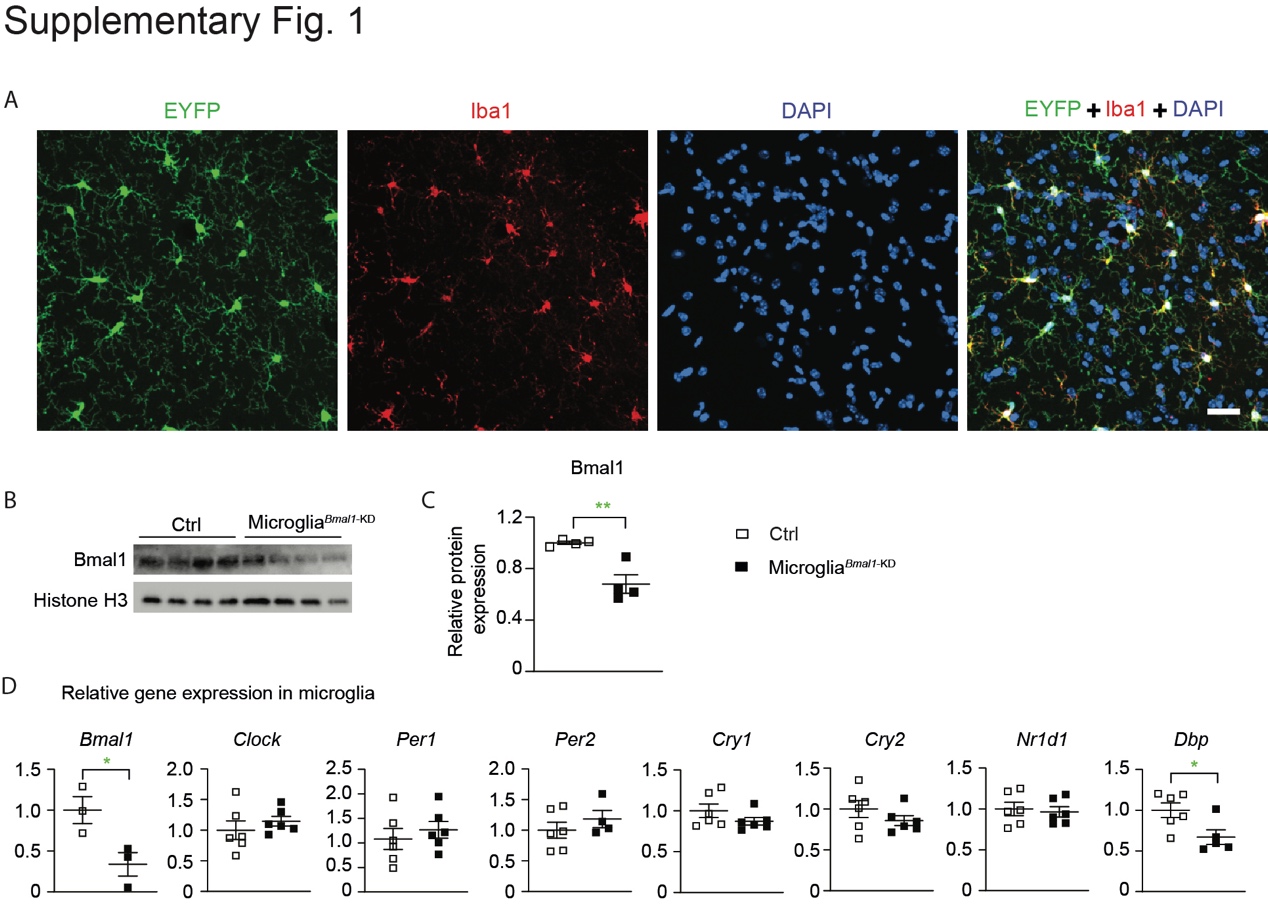


**Figure. S1. Microglia specific knock-down of Bmal1 (microglia*^Bmal1^*^-KD^) alters clock gene expression in adult female** **mice. (A)** Representative confocal images of EYFP, Iba1, and DAPI immunostaining in the hippocampus region of microglia*^Bmal1^*^-KD^ or Ctrl mice used in our study; scale bar, 30 µm. **(B** and **C)** Representative images and quantification of Western blotting showing immunoreactive Bmal1 and Histone H3 in microglia (n = 4 mice per group). **(D)** Expression of *Bmal1* and other clock genes in microglia isolated from the brain of Ctrl and microglia*^Bmal1^*^-KD^ female mice 3 weeks after the tamoxifen injection (n = 3-6 mice per group). Data are presented as means ± s.e.m. Green-colored asterisks * indicate a genotype effect; * *P* < 0.05, ** *P* < 0.01.

**
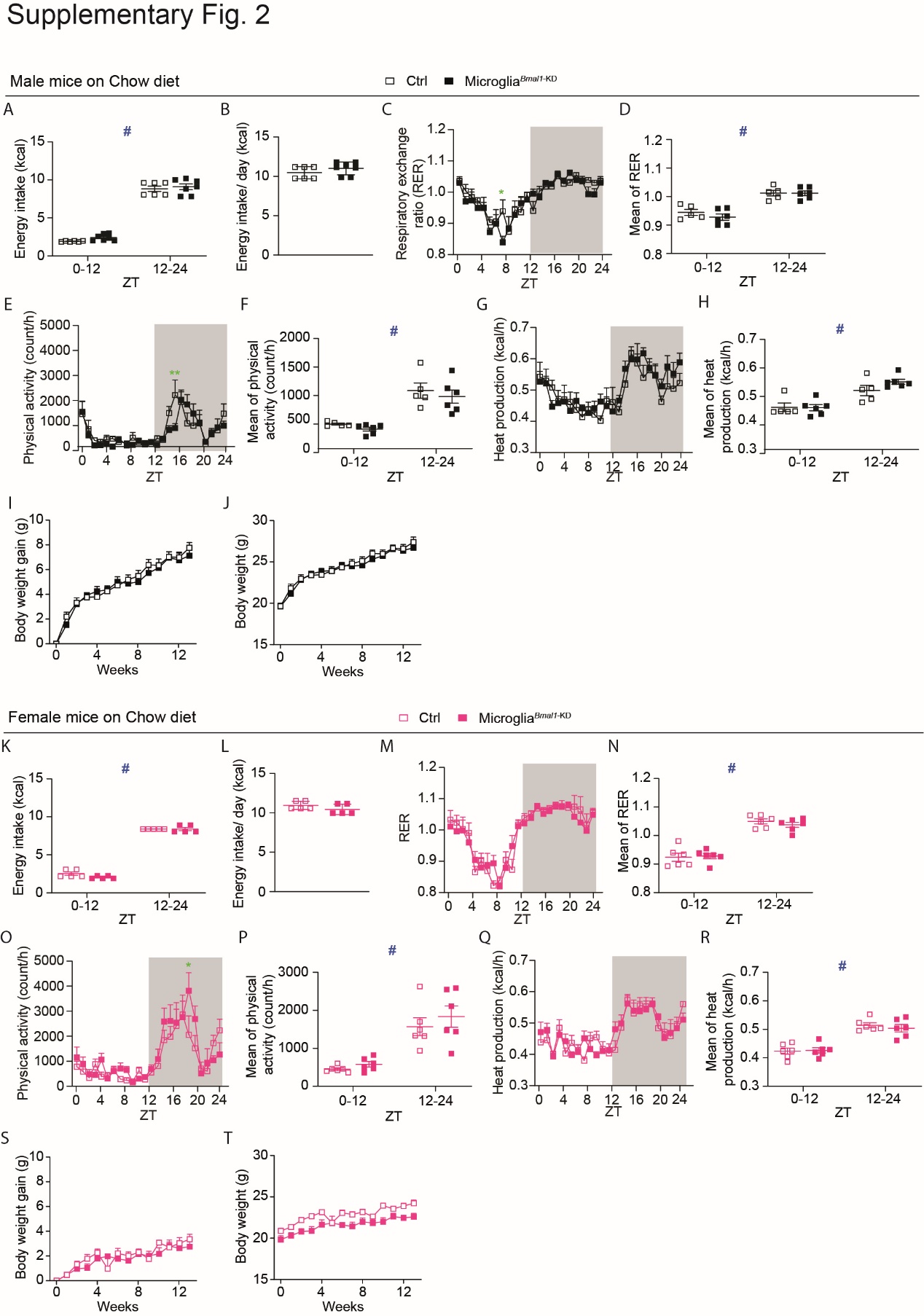
Supplemental Figure. 2**

**Figure. S2. Metabolic phenotype of Ctrl and microglia*^Bmal1^*^-KD^** **male and female** **mice on a standard chow diet. (A** to **J)** Metabolic data from male mice. **(A** and **B)** Energy intake during light and dark phase, and per day (n = 6-7 mice per group). **(C** and **D)** RER and the mean of RER during the light phase and dark phase (n = 5-6 mice per group). **(E** and **F)** Physical activity and the mean of physical activity during the light phase and dark phase (n = 5-6 mice per group). **(G** and **H)** Heat production and the mean of heat production during the light phase and dark phase (n = 5-6 mice per group). **(I** and **J)** The body weight gain and body weight. **(K** to **T)** Metabolic data from female mice. **(K** and **L)** Energy intake during light and dark phase and per day (n = 5-6 mice per group). **(M** and **N)** RER and the mean of RER during the light phase and dark phase (n = 6 mice per group). **(O** and **P)** Physical activity and the mean of physical activity during the light phase and dark phase (n = 6 mice per group). **(Q** and **R)** Heat production and the mean of heat production during the light phase and dark phase (n = 6 mice per group). **(S** and **T)** The body weight gain and body weight. Data are presented as means ± s.e.m. Green-colored asterisks * indicate a genotype effect; **#** for ZT0-12 compared to ZT12-24. * *P* < 0.05, ** *P* < 0.01, and **#** *P* < 0.05.


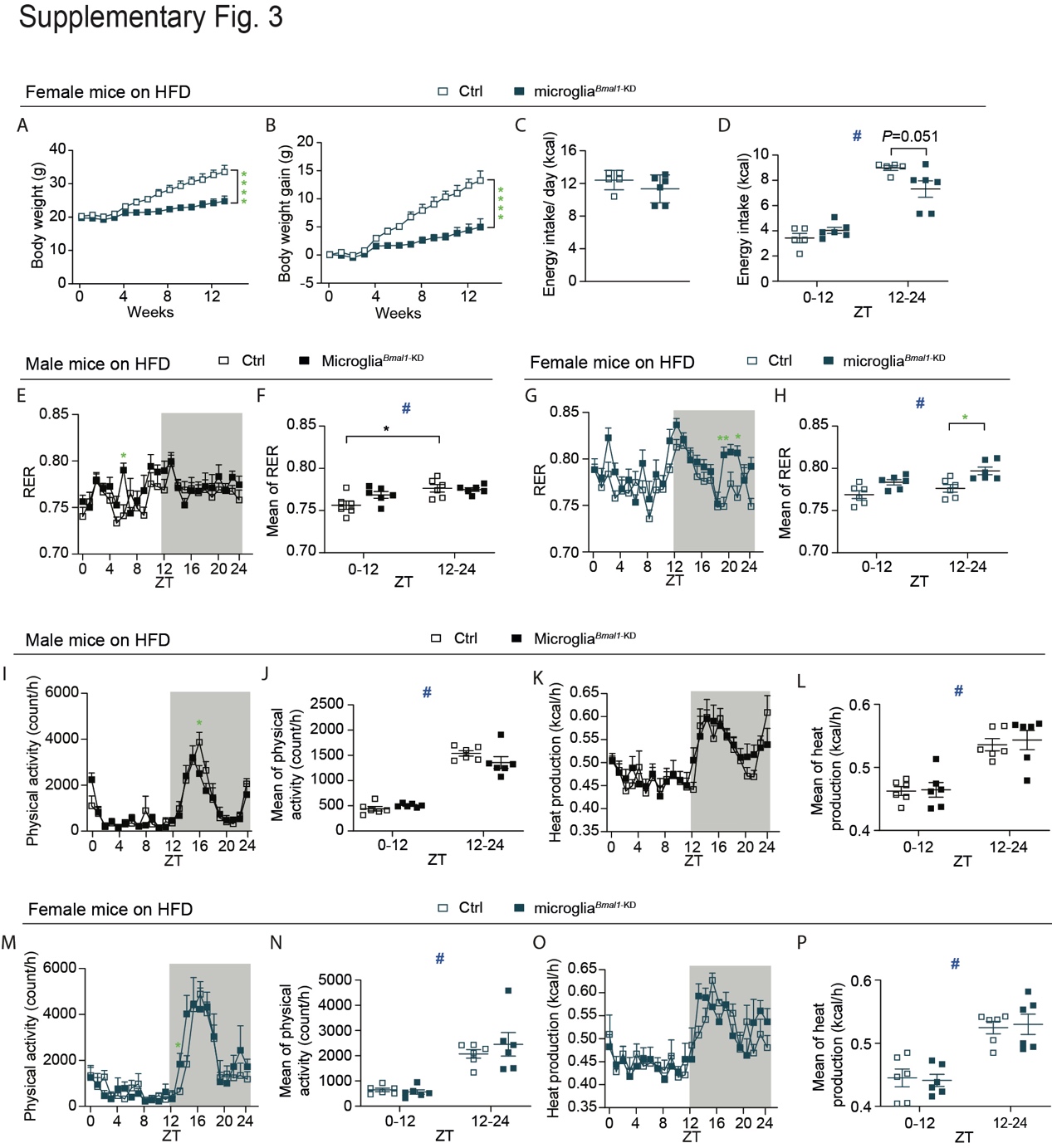
 **Supplemental Figure. 3**

**Figure. S3. Metabolic phenotype of Ctrl and microglia*^Bmal1^*^-KD^** **male and female mice** **on the HFD. (A** to **J)** Metabolic parameters of female mice fed with a HFD. **(A** and **B)** Body weight and body weight gain (n = 5-6 mice per group). **(C** and **D)** Energy intake of Ctrl and microglia*^Bmal1^*^-KD^ mice (n = 5-6 mice per group). **(E** and **F)** RER and the mean of RER during the light phase and dark phase of male mice fed a HFD (n = 6 mice per group). **(G** and **H)** RER and the mean of RER during the light phase and dark phase of female mice fed a HFD (n = 6 mice per group). **(I** to **L)** Metabolic data of male mice fed a HFD. **(I** and **J)** Physical activity and the mean of physical activity during the light phase and dark phase (n = 6 mice per group). **(K** and **L)** Heat production and the mean of heat production during the light phase and dark phase (n = 6 mice per group). **(M** to **P)** Metabolic data of female mice fed a HFD. **(M** and **N)** Physical activity and the mean of physical activity during the light phase and dark phase (n = 6 mice per group). **(O** and **P)** Heat production and the mean of heat production during the light phase and dark phase (n = 6 mice per group). Data are presented as means ± s.e.m. Green-colored asterisks * indicate a genotype effect; **#** for ZT0-12 compared to ZT12-24. * *P* < 0.05, ** *P* < 0.01, **** *P* < 0.0001, and **#** *P* < 0.05.

**Supplemental Figure. 4
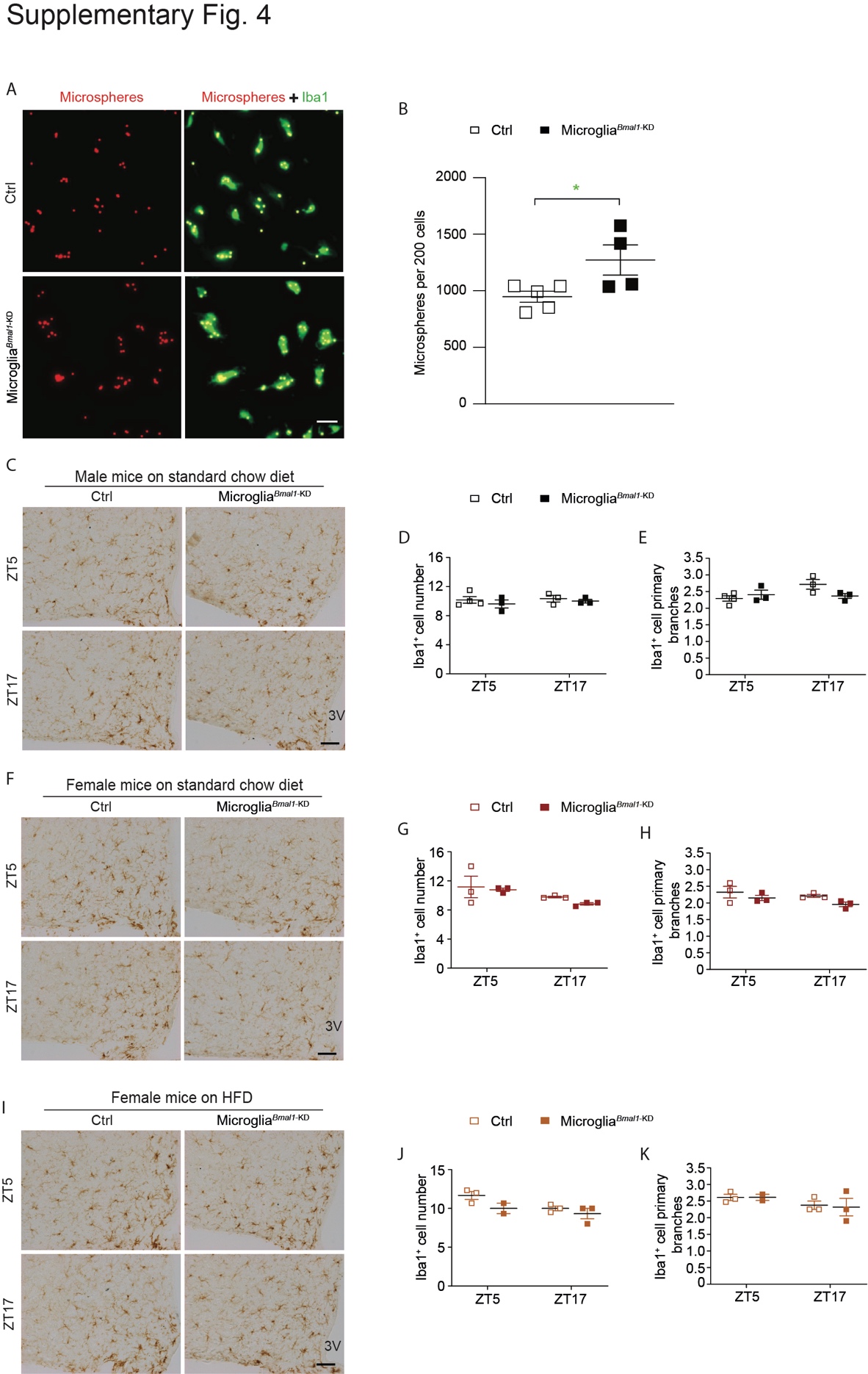
**

**Figure. S4. Microglial phagocytic capacity as well as cell number and primary projections in Ctrl and microglia*^Bmal1^*^-KD^** **mice** **on chow diet and in response to HFD. (A)** Image of microspheres in primary microglial cells in Ctrl and Bmal1-KD groups (n = 4-5 samples per group; 200 cells were counted in each sample). Scale bar, 20 µm. (**B**) Microspheres per sample (the average of microspheres per cell: 4.7 microspheres in Ctrl group; 6.4 microspheres in Bmal1-KD group). (**C**) Representative images of Iba1 immunostaining of male mice fed a standard chow diet. **(D** and **E)** Quantification of Iba^+^ microglial cell number and primary projections (n = 3 mice per group). **(F** to **H)** Representative images and quantification of Iba1^+^ microglial cell number and primary projections of female mice fed a standard chow diet (n = 3 mice per group). **(I** to **K)** Representative images and quantification of Iba1^+^ microglial cell number and primary projections of female mice fed a HFD (n = 2-3 mice per group). 3V, third ventricle. Scale bar, 100 µm. Data are presented as means ± s.e.m.

**Supplemental Figure. 5**
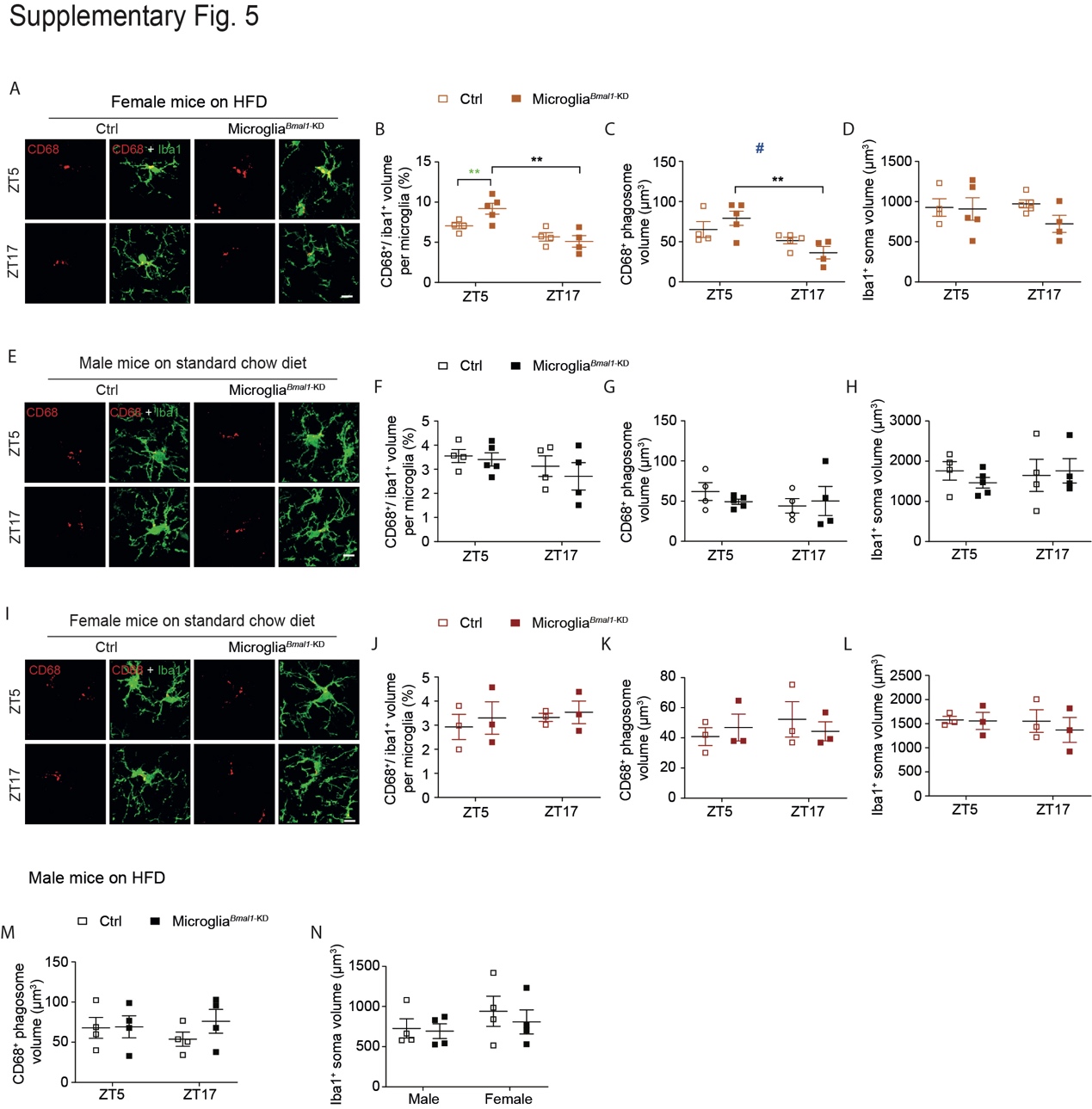


**Figure. S5. Microglial CD68**^+^**/ Iba1**^+^ **ratio in mice on a HFD and chow diet. (A)** Confocal images of CD68^+^ and Iba1^+^ in ARC of female mice fed a HFD (n = 4-5 mice per group). **(B** to **D)** Quantitative analyses of the percentage of CD68^+^ volume per microglia, CD68^+^ phagosome volume, and Iba1^+^ soma volume in female mice fed a HFD (100-125 cells were analyzed in each group). **(E)** Confocal images of CD68^+^ and Iba1^+^ in ARC of male mice fed a chow diet (n = 4-5 mice per group). **(F** to **H)** Quantitative analyses of the percentage of CD68^+^ volume per microglia, CD68^+^ phagosome volume, and Iba1^+^ soma volume in male mice fed a chow diet (100-125 cells were analyzed in each group). **(I)** Confocal images of CD68^+^ and Iba1^+^ in ARC of female mice fed a chow diet (n = 3 mice per group). **(J** to **L)** Quantitative analyses of the percentage of CD68^+^ volume per microglia, CD68^+^ phagosome volume, and Iba1^+^ soma volume in female mice fed a chow diet (75 cells were analyzed in each group). **(M** to **N)** Quantitative analyses of CD68^+^ phagosome volume and Iba1^+^ soma volume in male mice fed a HFD (n= 4-5 mice per group). Scale bar, 10 µm. Data are presented as means ± s.e.m. Green-colored asterisks * indicate a genotype effect; **#** when ZT5 is compared to ZT17. ** *P* < 0.01.


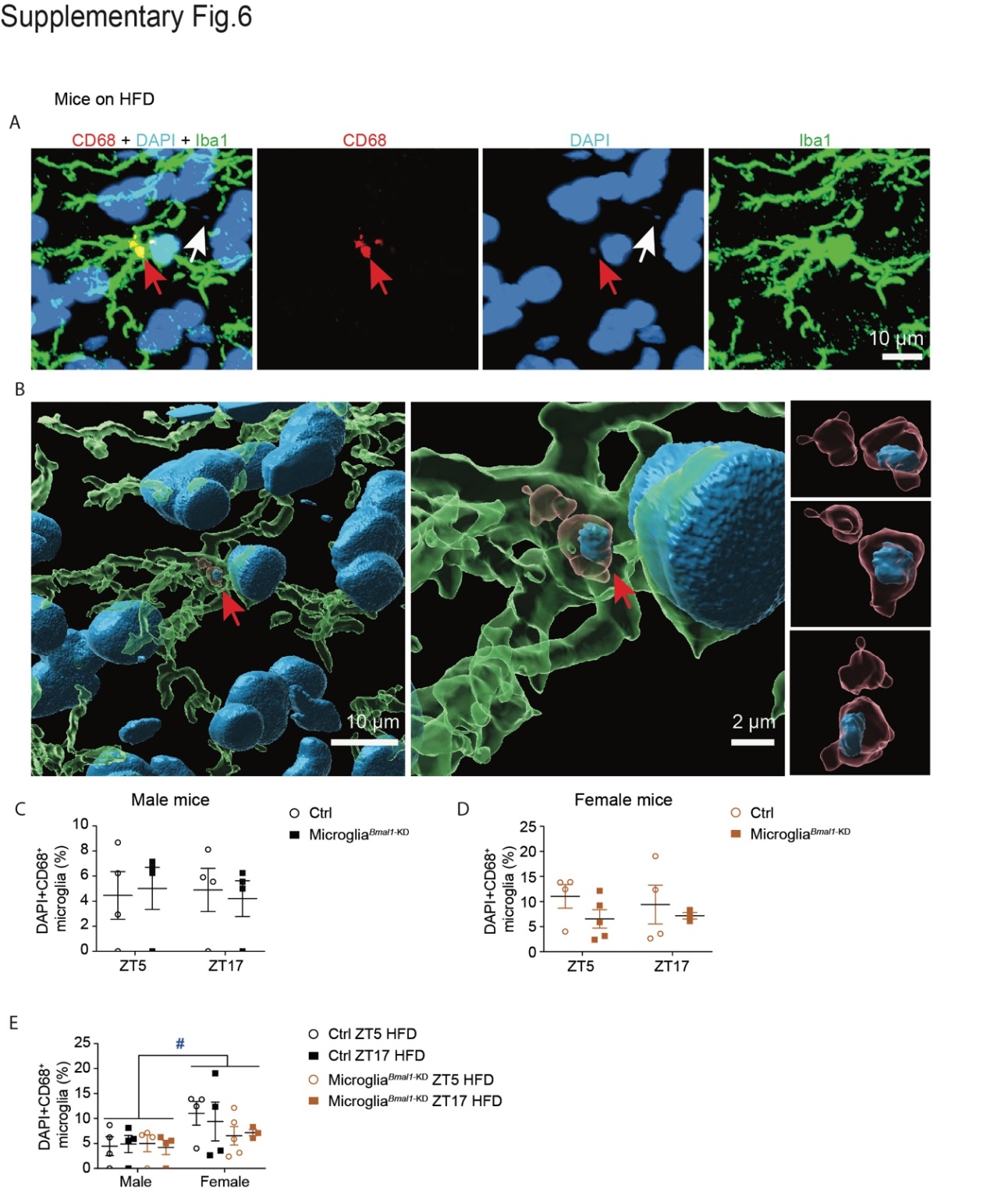
**Supplemental Figure. 6**

**Figure. S6. CD68, DAPI, and Iba1 triple-staining** **in the ARC of HFD-fed male and female mice. (A)** Confocal images of DAPI (blue), CD68 (red), and iba1 (green) triple labeling. The yellow color in the most left panel marks microglial phagosomes; the red arrow indicates the DAPI^+^ particle inside the CD68^+^ phagosome; the white arrow indicates the DAPI^+^ particle outside of the CD68^+^ phagosome. **(B)** 3D reconstruction of the confocal images in **A,** with surfacing of DAPI (solid blue), CD68 (transparent red), and iba1 (transparent green), and amplified side-scatter views**.** (**C** to **E**) Quantification of the percentage of microglia with DAPI^+^CD68^+^ phagosomes in male and female mice fed a HFD (n = 4-5 mice per group, 30-40 cells per mice). Data are presented as means + s.e.m. # *P* < 0.05.

**Supplemental Figure. 7**


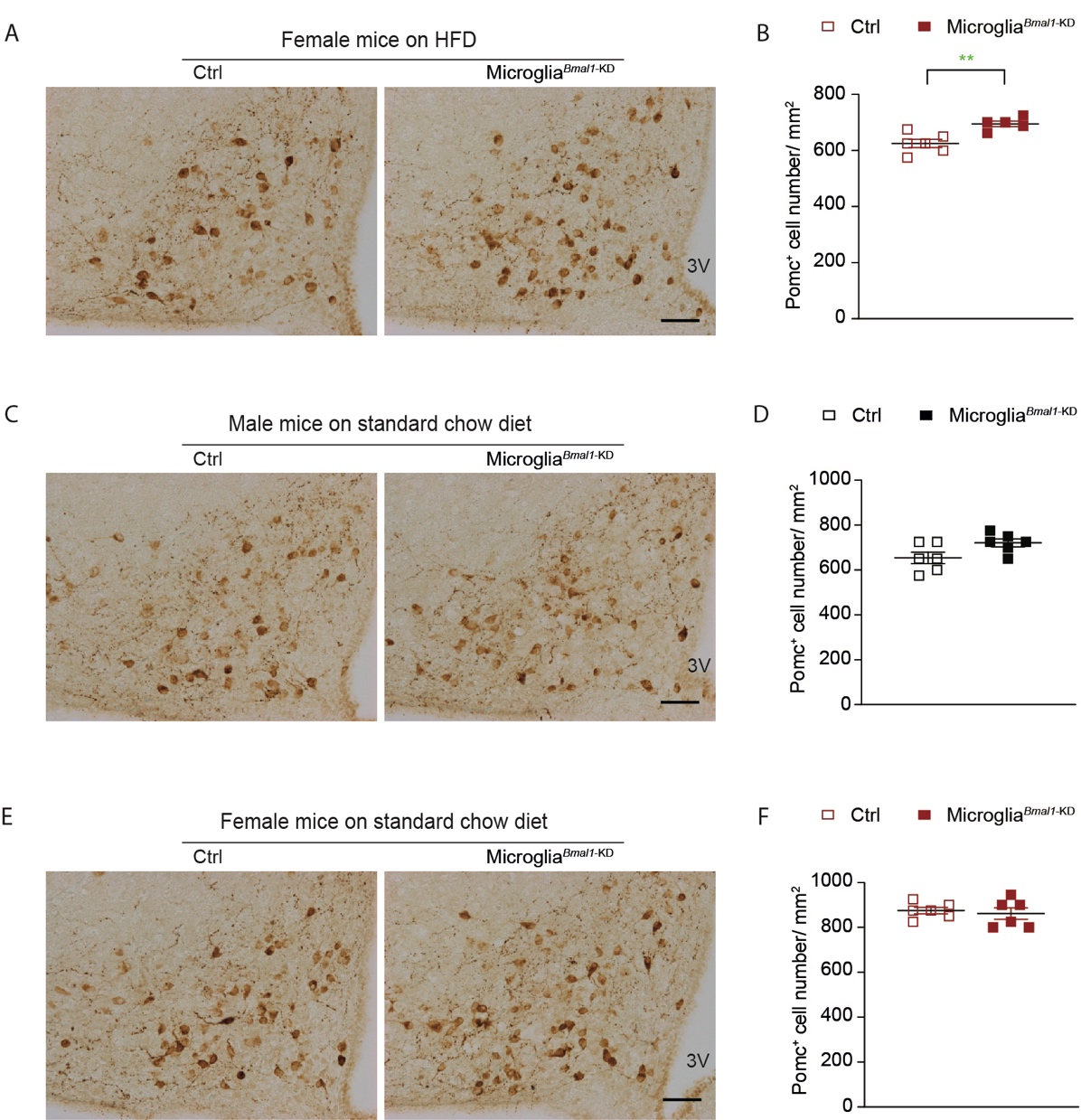


**Figure. S7. POMC^+^ neurons in Ctrl and microglia*^Bmal1^*^-KD^** **mice, males and females, on a HFD or standard chow diet. (A** and **B)** Images and quantification of POMC**^+^** neuronal cell number in female mice on a HFD (n = 5-6 mice per group). **(C** and **D)** Images and quantification of POMC**^+^** neurons in male mice on a chow diet (n = 6 mice per group). **(E** and **F)** Images and quantification of POMC**^+^** neurons in female mice on a chow diet (n = 6 mice per group). 3V, third ventricle. Scale bar, 100 µm. Data are presented as means ± s.e.m. Green-colored asterisks * indicate a genotype effect; ** *P* < 0.01.

**Supplemental Figure. 8**


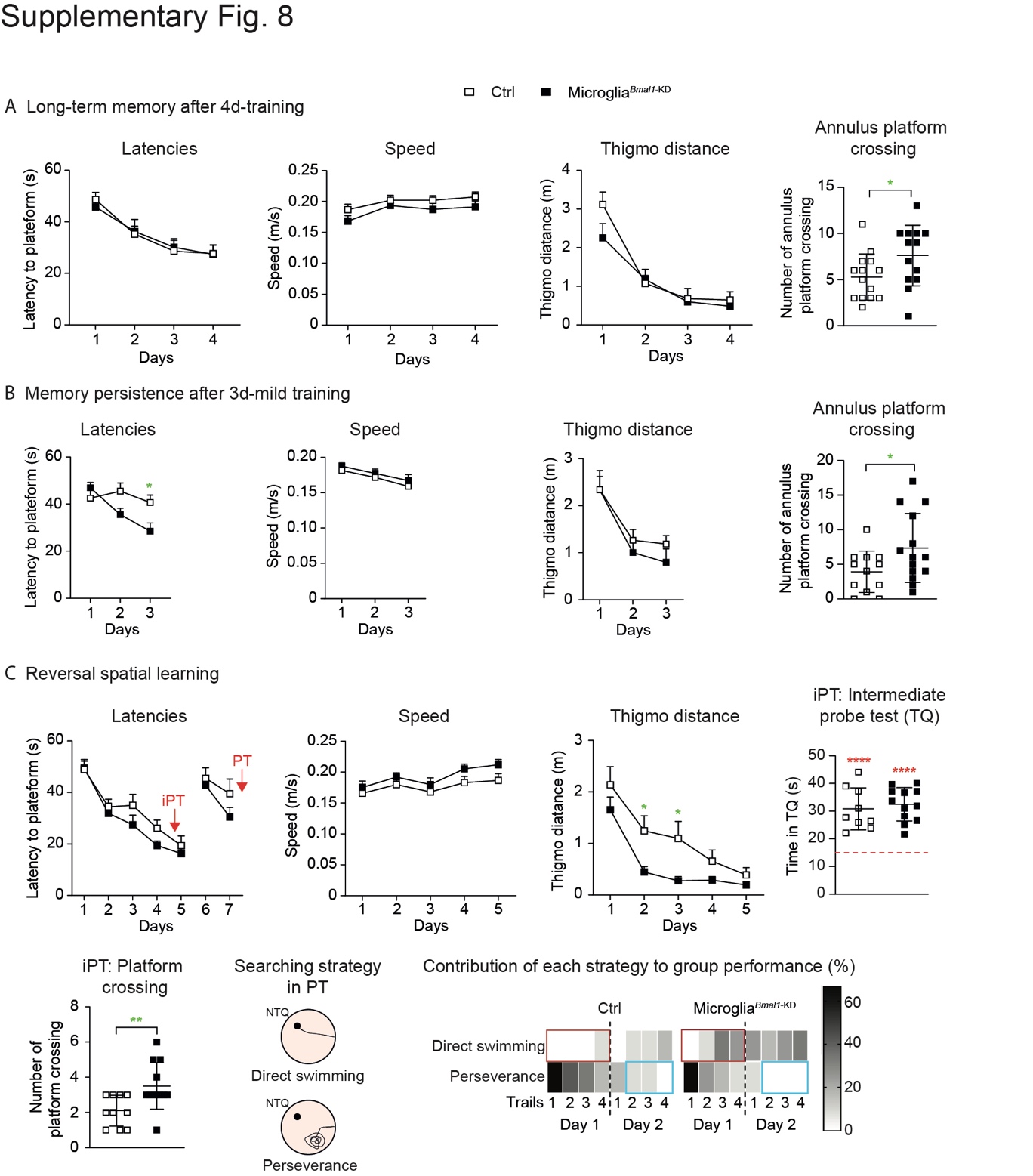


**Figure. S8. Behavioral evaluation of Ctrl and microglia*^Bmal1^*^-KD^** **mice. (A)** Parameters during 4 days MWM acquisition training and probe test (PT) (n = 13-14 mice per genotype). Related to Fig. 3C-E. **(B)** Parameters during 3 days MWM acquisition training and probe test (n = 12-14 mice per genotype). Related to Fig. 3F-H. **(C)** Parameters during 5 days of MWM acquisition training, 2 days of reversal training, and probe tests (n = 9-12 mice per genotype). Related to Fig. 3I-K. iPT, intermediate probe test, on day 5; PT, after 24 h of the last reversal training on day 8. Data are presented as means ± s.e.m. Green-colored asterisks * indicate a genotype effect; red-colored asterisks * indicate a comparison versus random (red line). * *P* < 0.05, ** *P* < 0.01, and **** *P* < 0.0001.

**Supplemental Figure. 9**


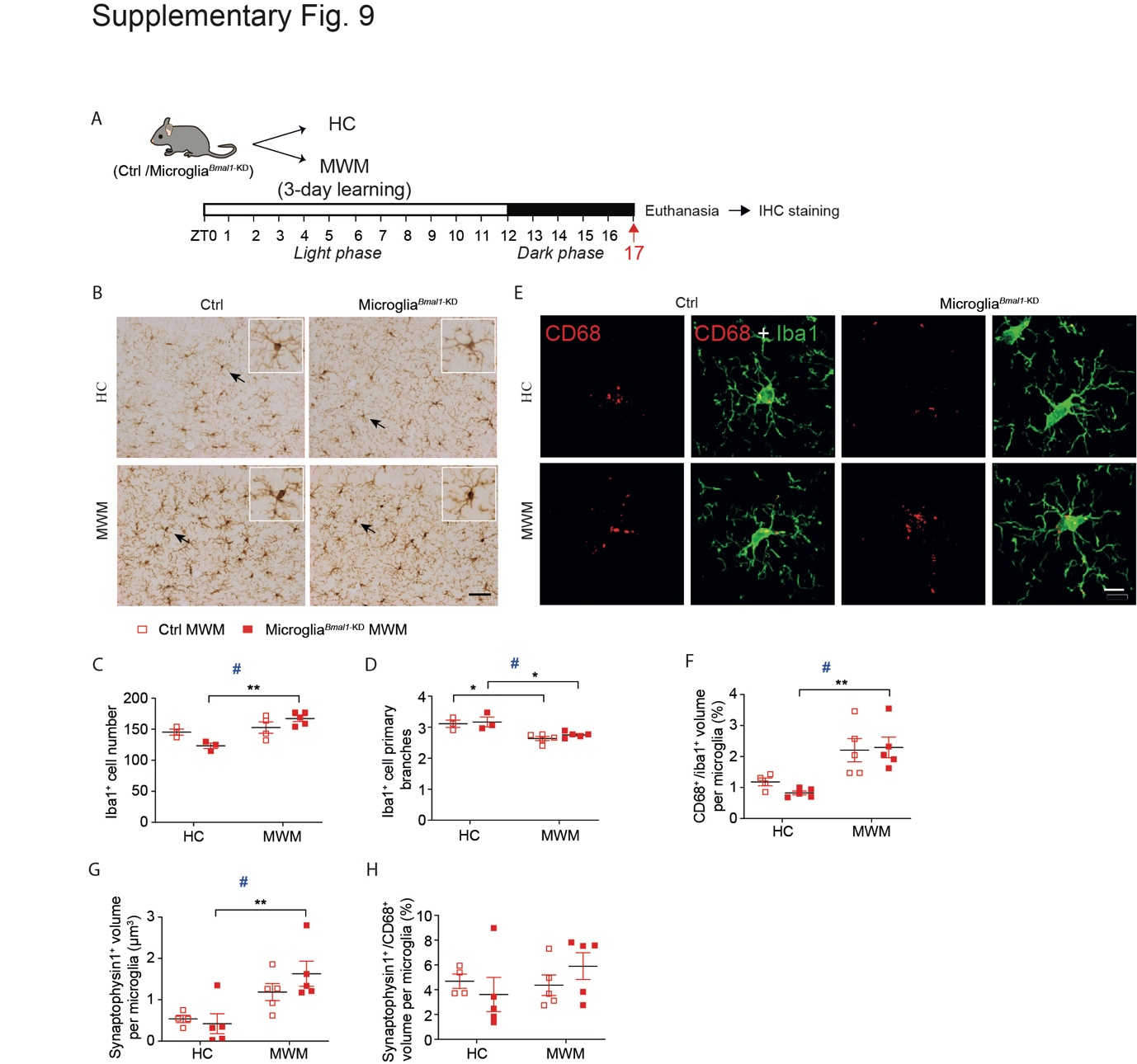


**Figure. S9. Microglial phagocytic capacity in the hippocampal stratum radiatum during the dark phase after learning. (A)** Experimental strategy. Mice received 3 days training in the MWM and were sacrificed at ZT17 of day 3. **(B)** Microglial immune reactivity in hippocampal CA1. Scale bar, 100 µm. **(C** and **D)** Quantification of Iba1^+^ microglial cell number and primary branches in home-cage (HC, n = 3 mice per group) and MWM (n = 4-5 mice per genotype) in CA1. **(E)** Confocal images of CD68 and Iba1 immunostaining in hippocampal stratum radiatum. Scale bar, 10 µm. **(F)** Quantitative analyses of the percentage of CD68^+^ volume per microglia in hippocampal stratum radiatum (HC, n = 4-5 mice per group; MWM, n = 5 mice per group; 100-125 cells were analyzed in each group). **(G** and **H)** Quantification of the volume of synaptophysin1^+^ in the CD68^+^ and the ratio of synaptophysin 1^+^/ CD68^+^ volume per microglia in hippocampal stratum radiatum (HC, n = 4-6 mice per group; MWM, n = 5 mice per group). Data are presented as means ± s.e.m. **#** when HC is compared to MWM. * *P* < 0.05, ** *P* < 0.01, and **#** *P* < 0.05.

**Supplemental Figure. 10**


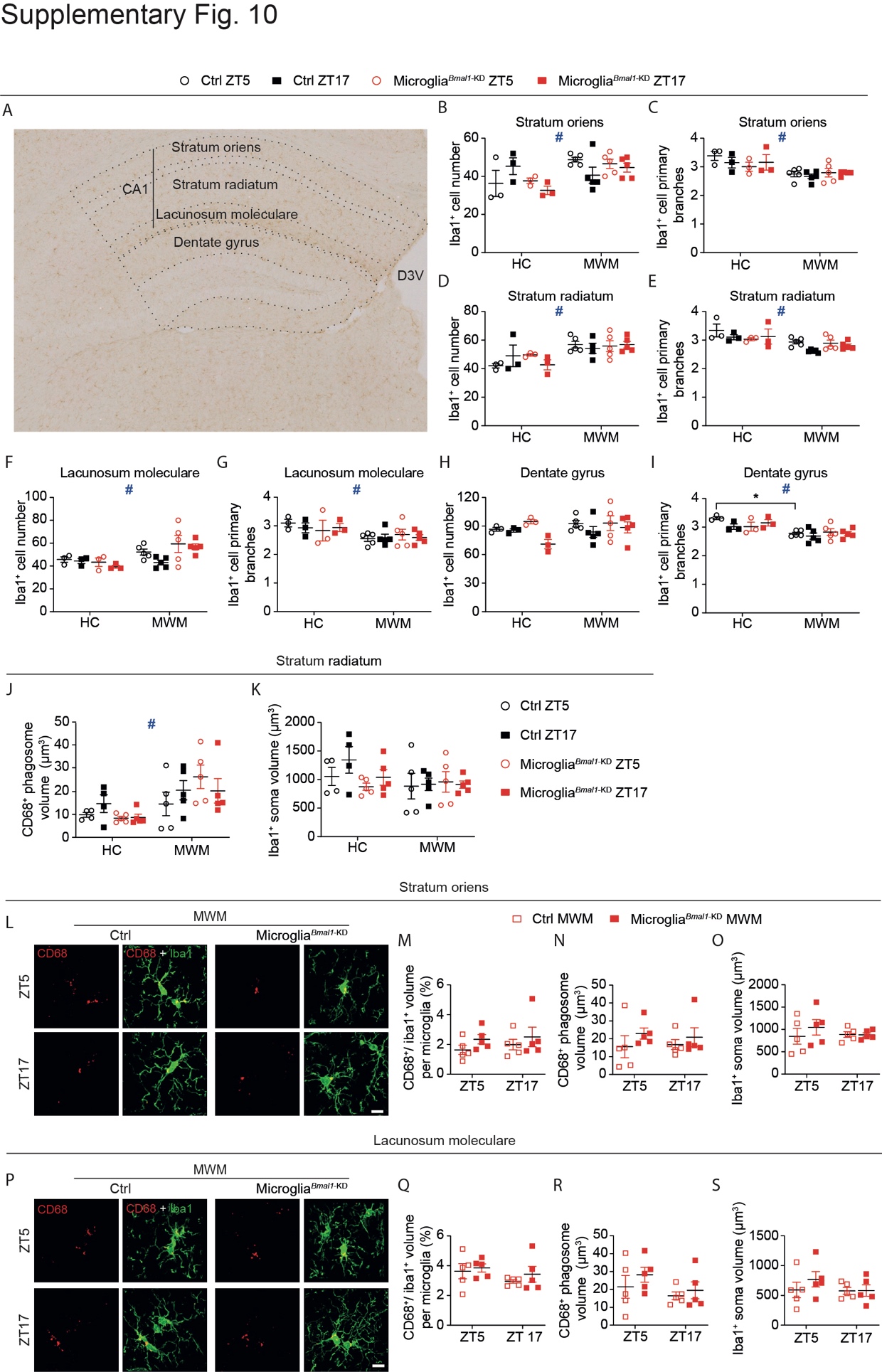


**Figure. S10. Microglial Iba1 and CD68/ Iba1 immunoreactivity in different hippocampal regions of Ctrl and microglia*^Bmal1^*^-KD^** **mice in the HC and MWM group at ZT5 and ZT17.** **(A)** Delineation of the area of the different hippocampal regions. D3V, dorsal third ventricle. **(B** to **I)** The microglial (Iba1^+^ cells) number and primary branches after 3 days MWM training and in the HC group in different layers of the mouse hippocampus (HC, n = 3 mice per group; MWM, n = 5 mice per group). **(J** and **K)** Quantitative analyses of the volume of CD68^+^ and Iba1^+^ in the hippocampal stratum radiatum in HC and MWM groups (HC, n = 3 mice per group; MWM, n = 5 mice per group). **(L)** Representative images of Iba1 and CD68 immunostaining in the hippocampal stratum oriens of the MWM group at ZT5 and ZT17. **(M** and **O)** Quantification of CD68 and Iba1 after 3 days of MWM training at ZT5 and ZT17 (n = 5 mice per group; 125 cells were analyzed in each group). **(P)** Representative images of Iba1 and CD68 immunostaining in the hippocampal lacunosum moleculare in the MWM group at ZT5 and ZT17. Scale bar: 10 µm. **(Q** and **S)** Quantification of CD68 and Iba1 in the hippocampal lacunosum moleculare after 3 days of MWM training at ZT5 and ZT17 (n = 5 mice per genotype; 125 cells were analyzed in each group). Scale bar, 10 µm. Data are presented as means ± s.e.m. **#** when HC is compared to MWM. * *P* < 0.05, and **#** *P* < 0.05.

**References**

1. Gao YQ, Vidal-Itriago A, Kalsbeek MJ, Layritz C, Garcia-Caceres C, Tom RZ, et al. Lipoprotein Lipase Maintains Microglial Innate Immunity in Obesity. *Cell Reports*. 2017; 20:3034.

2. Chatterjee S, Cassel R, Schneider-Anthony A, Merienne K, Cosquer B, Tzeplaeff L, et al. Reinstating plasticity and memory in a tauopathy mouse model with an acetyltransferase activator. *Embo Molecular Medicine*. 2018; 10.

3. Restivo L, Vetere G, Bontempi B, Ammassari-Teule M. The Formation of Recent and Remote Memory Is Associated with Time-Dependent Formation of Dendritic Spines in the Hippocampus and Anterior Cingulate Cortex. *Journal of Neuroscience*. 2009; 29:8206.

4. Wang XL, Wolff SEC, Korpel N, Milanova I, Sandu C, Rensen PCN, et al. Deficiency of the Circadian Clock Gene Bmal1 Reduces Microglial Immunometabolism. *Frontiers in Immunology*. 2020; 11.
